# Supplementary material for: Full genome characterization of 12 citrus tatter leaf virus isolates for the development of a detection assay
Source: PLoS One. 2019 Oct 17;14(10):e0223958. doi: 10.1371/journal.pone.0223958 (PMC6797102; doi:10.1371/journal.pone.0223958)
Supplement: S1 Fig — Multiple nucleotide sequences alignment of citrus tatter leaf virus and apple stem grooving virus isolated from citrus and citrus relatives host. Citrus tatter leaf virus detection assay targeting region (highlighted in dark grey) and primers-probe set are also shown. Apple stem grooving virus isolate P-209 is used here to represent the species. (PDF) [file pone.0223958.s001.pdf]

S1 Table. Oligonucleotide primers used in this study

Primer Name	Primer Sequence 5'- 3'	Nucleotide Position[1]	Note
CTLV-Outer-1	CTGAGACCAATCACTCTATCTCTG	469-493	5' Outer CTLV gene specific primer, for CTLV isolates TL100, TL101, TL102, TL103, TL110, and TL111
CTLV-Outer-2	CTAAGACCAATCACTCTACTTCTA	469-493	5' Outer CTLV gene specific primer, for CTLV isolates TL115 and IPPN122
CTLV-Outer-3	CTAAGACCAATCACTCTATCTCTA	469-493	5' Outer CTLV gene specific primer, for CTLV isolates TL104
CTLV-Outer-4	CAAGCCAATCACTCTGTCTCTG	469-493	5' Outer CTLV gene specific primer, for CTLV isolates TL112, TL113, and TL114
CTLV-Inner-1	GGATGGGAATGTGACTTGAATC	222-244	5' Inner CTLV gene specific primer, for CTLV isolates IPPN122, TL100, TL101, TL102, TL103, TL110, and
CTLV-Inner-2	GGATGGGAGTGTGACTTAAATC	222-244	5' Inner CTLV gene specific primer, for CTLV isolates TL104 and TL115
CTLV-Inner-3	GGATGAGAATGTGATTTAAATCCAATTGG	222-244	5' Inner CTLV gene specific primer, for CTLV isolates TL112, TL113, and TL114
CTLV-CP-Seq-5589-F	GRAAAGAGAGGRTTTAGGTCCCTCTCRGC	5589-5617	3' RACE CTLV gene specific primer

[1.] Nucleotide Position is base on reference genome of citrus tatter leaf virus isolate TL100 (NCBI GenBank Accession No. MH108975).
