# Supplementary material for: Full genome characterization of 12 citrus tatter leaf virus isolates for the development of a detection assay
Source: PLoS One. 2019 Oct 17;14(10):e0223958. doi: 10.1371/journal.pone.0223958 (PMC6797102; doi:10.1371/journal.pone.0223958)
Supplement: S2 Table — (PDF) [file pone.0223958.s003.pdf]

S2 Table. Full-length nucleotide sequence identities (%) of citrus tatter leaf virus isolates in this study and capilloviruses from NCBI GenBank database.

Isolate	Host		Geographic Origin	Isolation Year	GenBank Accesssion Number	GenBank Deposit Year	Clade	Cluster
AGSV-YTG	Apple	*Malus domestica*	China	2012	KJ579253	2014	A	I
ASGV-HH	Pear	*Pyrus pyrifolia* cv. 'Huanghua'	China	2009	JN701424	2012		
ASGV-CHN	Apple	*Malus domestica*	China	2011	JQ308181	2013		
ASG-241KP	Apple	*Malus domestica*	Japan	1992	D14995	2008		
ASGV-P-209	Apple	*Malus domestica*	Japan	1993	NC001749	2018		
ASGV-Nagami	Kumquat	*Fortunella margarita* (Lour.) Swing.	Japan	2016	LC184612	2017		
CTLV-ASGV-2-HJY	Citrus- Huang Jin Mi You	*Citrus maxima* (Burm.) Merrill	China	2016	MH144343	2018		
CTLV-MTH	Citrus- Ponkan Mandarin	*Citrus reticulata* Blanco	China	2013	KC588948	2013		
CTLV-IPPN122	Citrus- Sa Tou Satsuma	*Citrus unshiu* (Macf.) Marc.	China	1992	MH108986	2018		
CTLV-L	Lily	*Lilium longiflorum*	Japan	1993	D16681	2008		II
ASGV-Li-23	Apple	*Malus domestica*	Japan	1997	AB004063	2000		
ASGV-FKSS2	Citrus	*Citrus junos* Sieb. ex Tanaka	Japan	2014	LC143387	2016		
ASGV-N297	Citrus- Satsuma	*Citrus unshiu* (Macf.) Marc.	Japan	1987	LC184610	2017		
ASGV-AC	Apple	*Malus domestica*	Germany	2009	JX080201	2012	B	III
ASGVp12	Apple	*Malus domestica* cv. Red Chief	India	2011	HE978837	2015		
ASGV-Ac	Actinidia	*Actinidia* sp.	China	2015	KX988001	2017		
ASGV-Matsuco	Citrus	*Citrus tamurana*	Japan	2014	LC084659	2015		
CTLV-Ponkan8	Citrus- Ponkan Mandarin	*Citrus reticulata* Blanco	Taiwan	2012	KY706358	2018		
CTLV-Pk	Citrus- Ponkan Mandarin	*Citrus reticulata* Blanco	Taiwan	2012	JX416228	2012		
CTLV-TL113	Citrus- Citron	*Citrus medica* L.	China	2014	MH108983	2018		
CTLV-TL114	Citrus- Citron	*Citrus medica* L.	China	2014	MH108984	2018		
CTLV-TL112	Citrus- Citron	*Citrus medica* L.	China	2014	MH108982	2018		
CTLV-LCd-NA-1	Citrus- Sweet Orange	*Citrus sinensis* L. Osb.	Taiwan	2004	FJ355920	2008		
CTLV-HJY	Citrus- Huang Jin Mi You	*Citrus maxima* (Burm.) Merrill	China	2016	MH144341	2018		
CTLV-Kumquat1	Kumquat	*Fortunella margarita* (Lour.) Swing.	Taiwan	2004	AY646511	2004		
CTLV-Shatang Orange	Citrus- Shatang Mandarin	*Citrus reticulata* Blanco	China	2011	JQ765412	2012		
CTLV-XHC	Citrus- Sweet Orange	*Citrus sinensis* L. Osb.	China	2013	KC588947	2013		
CTLV-ML	Citrus- Meyer Lemon	*Citrus limon* (L.) Burm.f. hyb.	FL, USA	2008	EU553489	2010		IV
CTLV-TL111	Citrus- Meyer Lemon	*Citrus limon* (L.) Burm.f. hyb.	FL, USA	1964	MH108981	2018		
CTLV-TL110	Citrus- Little Sweetie Satsuma	*Citrus unshiu* (Macf.) Marc.	CA, USA	1989	MH108980	2018		
CTLV-TL103	Citrus- Hirado Buntan Pummelo	*Citrus maxima* (Burm.) Merrill	Japan	1983	MH108978	2018		
CTLV-TL101	Citrus- Meyer Lemon	*Citrus limon* (L.) Burm.f. hyb.	CA, USA	1969	MH108976	2018		
CTLV-TL100	Citrus- Meyer Lemon	*Citrus limon* (L.) Burm.f. hyb.	TX, USA	1958	MH108975	2018		
CTLV-TL102	Citrus- Meyer Lemon	*Citrus limon* (L.) Burm.f. hyb.	CA, USA	1958	MH108977	2018		
CTLV-TL104	Citrus- Kobeni Mikan Tangor	*Citrus reticulata* x *Citrus sinensis*	China	1987	MH108979	2018		
CTLV-TL115	Citrus- Dekopon Tangor	*Citrus reticulata* x *Citrus sinensis*	Japan	2007	MH108985	2018		
ASGV-Kiyomi	Citrus	*Citrus unshiu* x *Citrus sinensis*	Japan	2016	LC184611	2017		
CTLV-ASGV-1-HJY	Citrus- Huang Jin Mi You	*Citrus maxima* (Burm.) Merrill	China	2016	MH144342	2018		
PBNLSV	Pear	*Pyrus pyrifolia*	South Korea	2004	AY596172	2004		Outgroup
ASGV-kfp	Pear	*Pyrus pyrifolia*	China	2014	KR106996	2015		
AGSV-HT	Apple	*Malus* spp. Crabapple	China	2015	KU947036	2017		
CVA	Cherry	*Prunus avium* L. cv. Sam	Germany	1994	NC003689	2018		

Isolate	AGSV-YTG	ASGV-HH	ASGV-CHN	ASG-241KP	ASGV-P-209	ASGV-Nagami	CTLV-ASGV-2-HJY	CTLV-MTH	CTLV-IPPN122	CTLV-L	ASGV-Li-23	ASGV-FKSS2	ASGV-N297	ASGV-AC
AGSV-YTG														
ASGV-HH	84.52													
ASGV-CHN	83.23	90.54												
ASG-241KP	86.78	87.12	86.34											
ASGV-P-209	86.78	87.12	86.34	100.0										
ASGV-Nagami	87.06	87.06	85.92	92.96	92.96									
CTLV-ASGV-2-HJY	86.26	87.23	85.97	92.36	92.36	93.02								
CTLV-MTH	86.63	86.49	85.78	91.07	91.07	91.42	90.84							
CTLV-IPPN122	84.52	84.57	83.72	86.06	86.06	86.54	87.23	86.82						
CTLV-L	83.04	84.09	83.12	82.98	82.98	82.95	83.11	83.45	83.48					
ASGV-Li-23	82.78	84.08	83.03	82.97	82.97	82.97	83.17	83.27	83.41	98.47				
ASGV-FKSS2	83.37	84.89	83.54	83.44	83.44	83.68	83.85	84.13	83.96	94.90	94.70			
ASGV-N297	82.98	84.43	83.35	83.27	83.27	83.68	83.68	84.06	83.38	94.24	94.04	95.27		
ASGV-AC	82.58	81.89	81.53	82.66	82.66	82.23	82.03	82.06	82.09	81.66	81.64	82.23	82.28	
ASGVp12	82.12	81.16	80.90	81.76	81.76	81.42	80.99	81.49	81.42	80.84	80.87	81.47	81.42	97.31
ASGV-Ac	82.06	81.35	81.50	81.89	81.89	82.14	81.91	81.83	82.23	81.61	81.49	82.20	82.09	82.31
ASGV-Matsuco	82.54	81.66	81.44	81.81	81.81	82.25	81.55	82.57	81.14	81.37	81.37	81.78	81.72	81.71
CTLV-Ponkan8	82.43	81.97	81.60	81.72	81.72	82.23	81.66	82.13	81.11	81.58	81.57	81.74	81.61	81.86
CTLV-Pk	82.43	81.97	81.60	81.72	81.72	82.23	81.66	82.14	81.12	81.57	81.57	81.74	81.61	81.86
CTLV-TL113	82.35	82.01	81.60	81.64	81.64	82.15	81.66	82.08	81.18	81.49	81.46	81.75	81.54	81.98
CTLV-TL114	82.26	81.83	81.40	81.93	81.93	82.29	81.98	82.60	81.51	81.52	81.47	81.91	81.71	81.97
CTLV-TL112	82.30	81.71	81.60	82.07	82.07	82.60	81.94	82.37	81.58	81.60	81.61	81.95	81.78	82.29
CTLV-LCd-NA-1	82.14	81.46	81.41	81.66	81.66	82.32	81.58	82.01	81.26	81.28	81.29	81.66	81.43	82.11
CTLV-HJY	82.24	81.55	81.29	81.89	81.89	82.29	81.68	82.29	81.35	81.15	81.17	81.40	81.25	81.81
CTLV-Kumquat1	82.41	81.54	81.20	81.87	81.87	82.17	81.74	82.38	81.37	81.43	81.30	81.65	81.55	81.83
CTLV-Shatang Orange	82.27	81.66	81.35	81.69	81.69	82.31	81.52	82.34	81.45	81.18	81.18	81.43	81.41	81.85
CTLV-XHC	81.89	81.06	80.90	81.33	81.33	81.80	81.23	81.89	80.88	80.91	80.89	81.11	81.09	81.83
CTLV-ML	81.95	81.12	81.52	81.64	81.64	82.27	81.33	82.09	82.18	81.38	81.27	81.66	81.61	81.77
CTLV-TL111	81.95	81.12	81.52	81.64	81.64	82.27	81.33	82.09	82.18	81.38	81.27	81.66	81.61	81.77
CTLV-TL110	81.95	81.12	81.52	81.64	81.64	82.27	81.33	82.09	82.18	81.38	81.27	81.66	81.61	81.77
CTLV-TL103	82.00	81.15	81.50	81.66	81.66	82.23	81.35	82.10	82.20	81.40	81.29	81.67	81.63	81.78
CTLV-TL101	81.99	81.29	81.47	81.61	81.61	82.18	81.30	82.10	82.18	81.25	81.16	81.61	81.53	81.95
CTLV-TL100	82.04	81.29	81.53	81.84	81.84	82.32	81.47	82.17	82.10	81.30	81.23	81.77	81.81	81.95
CTLV-TL102	82.23	81.35	81.64	81.95	81.95	82.40	81.67	82.18	82.40	81.49	81.40	81.93	81.83	81.98
CTLV-TL104	81.73	81.47	81.83	81.80	81.80	82.46	81.92	82.46	82.03	81.37	81.29	82.14	81.90	82.06
CTLV-TL115	81.50	81.15	81.27	81.87	81.87	82.18	81.61	82.35	81.81	81.49	81.29	82.03	81.73	81.93
ASGV-Kiyomi	81.58	81.32	81.40	81.92	81.92	82.30	81.67	82.40	81.90	81.49	81.29	82.06	81.86	82.23
CTLV-ASGV-1-HJY	82.60	81.86	81.50	82.49	82.49	82.54	82.34	82.32	82.35	82.00	81.92	82.29	82.37	82.68
PBNLSV	80.30	79.83	80.00	79.76	79.76	79.77	79.86	79.86	80.08	80.44	80.44	80.63	80.63	80.09
ASGV-kfp	79.98	79.65	79.50	79.64	79.64	80.13	79.92	79.98	79.76	79.58	79.52	79.93	80.01	80.06
AGSV-HT	80.87	79.34	78.90	79.09	79.09	79.41	79.09	79.65	79.29	78.94	78.82	79.23	79.07	79.61
CVA	26.57	26.69	26.20	26.89	26.89	26.70	26.30	26.61	26.33	25.73	25.86	25.91	25.76	26.16

Isolate	ASGVp12	ASGV-Ac	ASGV-Matsuco	CTLV-Ponkan8	CTLV-Pk	CTLV-TL113	CTLV-TL114	CTLV-TL112	CTLV-LCd-NA-1	CTLV-HJY	CTLV-Kumquat1	CTLV-Shatang Orange	CTLV-XHC	CTLV-ML
ASGV-Ac	81.86													
ASGV-Matsuco	81.55	82.23												
CTLV-Ponkan8	81.61	82.20	95.13											
CTLV-Pk	81.61	82.20	95.13	100.0										
CTLV-TL113	81.73	82.26	95.05	99.43	99.43									
CTLV-TL114	81.93	82.57	93.88	96.18	96.18	96.35								
CTLV-TL112	81.92	82.49	93.51	93.27	93.27	93.75	92.67							
CTLV-LCd-NA-1	81.72	82.43	94.24	93.95	93.93	94.47	93.42	98.73						
CTLV-HJY	81.69	82.52	94.19	93.88	93.88	93.81	94.75	94.11	95.01					
CTLV-Kumquat1	81.59	82.25	94.11	93.96	93.95	93.87	95.52	94.31	95.10	97.18				
CTLV-Shatang Orange	81.59	82.34	94.45	94.10	94.11	94.10	95.18	94.53	95.35	97.53	97.96			
CTLV-XHC	81.49	82.12	94.10	93.65	93.67	93.59	94.81	94.33	95.18	97.19	97.59	97.98		
CTLV-ML	81.42	81.50	81.86	81.84	81.84	81.98	81.93	82.04	81.90	81.98	81.86	81.72	81.43	
CTLV-TL111	81.42	81.50	81.86	81.84	81.84	81.98	81.93	82.04	81.90	81.98	81.86	81.72	81.43	100.0
CTLV-TL110	81.42	81.50	81.86	81.84	81.84	81.98	81.93	82.04	81.90	81.98	81.86	81.72	81.43	100.0
CTLV-TL103	81.44	81.52	81.87	81.86	81.86	82.00	81.95	82.06	81.92	82.00	81.87	81.73	81.44	99.95
CTLV-TL101	81.58	81.39	81.81	81.87	81.87	82.01	82.02	82.04	81.92	81.86	81.89	81.64	81.36	98.70
CTLV-TL100	81.55	81.55	81.86	81.93	81.93	82.07	82.03	82.12	81.98	81.97	81.89	81.69	81.41	98.52
CTLV-TL102	81.56	81.50	81.83	81.92	81.92	82.04	82.01	82.30	81.87	81.98	82.00	81.73	81.43	97.99
CTLV-TL104	81.58	81.97	81.78	82.03	82.03	82.10	82.40	82.00	81.81	81.86	81.95	81.70	81.47	92.51
CTLV-TL115	81.47	81.67	81.95	82.01	82.01	82.06	82.44	82.00	81.75	82.00	82.10	81.89	81.66	92.02
ASGV-Kiyomi	81.64	81.61	81.92	81.97	81.97	81.98	82.37	81.95	81.73	81.97	82.14	81.89	81.72	92.34
CTLV-ASGV-1-HJY	81.78	82.12	80.77	80.94	80.94	80.92	81.31	81.46	80.88	81.03	80.88	80.91	80.74	81.78
PBNLSV	79.88	80.43	79.40	79.03	79.04	78.95	79.37	79.23	78.95	79.41	79.01	78.99	78.82	79.39
ASGV-kfp	79.22	79.41	80.26	80.12	80.10	79.92	80.15	80.10	80.01	80.27	80.06	80.10	79.90	79.52
AGSV-HT	78.92	78.97	79.23	79.19	79.20	79.12	79.64	79.29	79.23	79.23	79.26	79.31	78.97	79.67
CVA	25.88	26.15	25.98	26.41	26.41	26.43	26.15	26.33	26.10	25.95	26.12	26.04	25.91	26.82

Isolate	CTLV-TL111	CTLV-TL110	CTLV-TL103	CTLV-TL101	CTLV-TL100	CTLV-TL102	CTLV-TL104	CTLV-TL115	ASGV-Kiyomi	CTLV-ASGV-1-HJY	PBNLSV	ASGV-kfp	AGSV-HT	CVA
CTLV-TL110	100.0													
CTLV-TL103	99.95	99.95												
CTLV-TL101	98.70	98.70	98.66											
CTLV-TL100	98.52	98.52	98.47	98.98										
CTLV-TL102	97.99	97.99	98.01	98.56	98.33									
CTLV-TL104	92.51	92.51	92.56	92.87	92.60	92.54								
CTLV-TL115	92.02	92.02	92.04	92.30	92.20	92.05	95.73							
ASGV-Kiyomi	92.34	92.34	92.39	92.65	92.50	92.34	95.95	98.70						
CTLV-ASGV-1-HJY	81.78	81.78	81.80	81.82	81.87	82.03	82.81	82.49	82.57					
PBNLSV	79.39	79.39	79.38	79.59	79.55	79.75	80.12	79.53	79.39	79.32				
ASGV-kfp	79.52	79.52	79.56	79.59	79.52	79.48	79.72	79.70	79.61	80.27	79.19			
AGSV-HT	79.67	79.67	79.69	79.68	79.79	79.81	79.44	79.47	79.41	78.78	78.43	79.21		
CVA	26.82	26.82	26.82	26.82	26.75	26.98	26.80	26.60	26.58	26.67	26.31	25.99	26.24	

Abbreviation: CTLV: citrus tatter leaf virus; ASGV: apple stem grooving virus; PBNLSV: pear black necrotic leaf spot virus; CVA: cherry virus A
