# Supplementary material for: Full genome characterization of 12 citrus tatter leaf virus isolates for the development of a detection assay
Source: PLoS One. 2019 Oct 17;14(10):e0223958. doi: 10.1371/journal.pone.0223958 (PMC6797102; doi:10.1371/journal.pone.0223958)
Supplement: S4 Table — (PDF) [file pone.0223958.s005.pdf]

S4 Table. Nucleotide sequence identities (%) of coat protein (CP) and 3'-untranslated region (3'-UTR).

Isolate	GenBank	CTLV-IPPN122	CTLV-TL100	CTLV-TL101	CTLV-TL102	CTLV-TL103	CTLV-TL104	CTLV-TL110	CTLV-TL111	CTLV-TL112	CTLV-TL113	CTLV-TL114	CTLV-TL115	CTLV-MTH	CTLV-XHC	CTLV-Pk	CTLV-Ponkan8	CTLV-ML	CTLV-Kumquat1	CTLV-LCd-NA-1	CTLV-Shatang Orange	CTLV-HJY	CTLV-ASGV-1-HJY	CTLV-ASGV-2-HJY	ASGV-Matsuco	ASGV-FKSS2	ASGV-N297	ASGV-Kiyomi	ASGV-Nagami	CTLV-L	ASGV-Li-23	ASGV-P-209	ASGVp12	ASGV-AC	ASGV-HH	ASGV-241KP	ASGV-kfp	ASGV-Ac	ASGV-CHN	ASGV-YTG	ASGV-HT	PBNLSV
CTLV-IPPN122	MH108986																																									
CTLV-TL100	MH108975	91.22																																								
CTLV-TL101	MH108976	91.22	99.53																																							
CTLV-TL102	MH108977	93.10	96.72	96.72																																						
CTLV-TL103	MH108978	91.57	98.83	98.83	96.49																																					
CTLV-TL104	MH108979	92.16	96.14	96.14	95.43	96.14																																				
CTLV-TL110	MH108980	91.46	99.18	99.18	96.37	99.64	95.78																																			
CTLV-TL111	MH108981	91.46	99.18	99.18	96.37	99.64	95.78	100.00																																		
CTLV-TL112	MH108982	93.22	91.11	91.11	93.92	91.22	92.51	91.11	91.11																																	
CTLV-TL113	MH108983	91.23	89.94	89.94	90.77	90.05	90.64	89.94	89.94	91.35																																
CTLV-TL114	MH108984	91.35	90.29	90.29	91.00	90.40	91.11	90.29	90.29	91.58	96.84																															
CTLV-TL115	MH108985	92.16	95.90	95.90	95.20	95.90	97.89	95.78	95.78	92.51	90.17	90.64																														
CTLV-MTH	KC588948	98.13	91.46	91.46	92.99	91.81	92.16	91.69	91.69	93.34	91.47	92.05	91.92																													
CTLV-XHC	KC588947	90.78	89.70	89.70	90.18	89.82	90.52	89.70	89.70	90.77	96.02	98.24	90.05	91.48																												
CTLV-Pk	JX416228	91.23	89.94	89.94	90.77	90.05	90.64	89.94	89.94	91.35	100.00	96.84	90.17	91.47	96.02																											
CTLV-Ponkan8	KY706358	91.13	89.94	89.94	90.77	90.05	90.64	89.94	89.94	91.35	100.00	96.84	90.17	91.36	95.91	100.00																										
CTLV-ML	EU553489	91.46	99.18	99.18	96.37	99.64	95.78	100.00	100.00	91.11	89.94	90.29	95.78	91.69	89.70	89.94	89.94																									
CTLV-Kumquat1	AY646511	91.24	90.29	90.29	91.00	90.40	91.11	90.29	90.29	91.58	96.84	100.00	90.64	91.94	98.13	96.84	96.84	90.29																								
CTLV-LCd-NA-1	FJ355920	91.24	90.17	90.17	90.65	90.29	91.46	90.17	90.17	91.47	96.49	97.42	90.76	91.59	97.19	96.49	96.49	90.17	97.43																							
CTLV-Shatang Orange	JQ765412	91.36	90.17	90.17	90.77	90.29	90.99	90.17	90.17	91.35	96.61	98.36	90.52	92.06	98.24	96.61	96.49	90.17	98.24	97.54																						
CTLV-HJY	MH144341	91.58	90.76	90.76	91.23	90.87	91.57	90.76	90.76	91.70	96.96	98.83	91.11	92.17	98.48	96.96	96.96	90.76	98.83	98.36	98.83																					
CTLV-ASGV-1-HJY	MH144342	92.28	89.82	89.82	91.47	90.17	91.46	90.05	90.05	94.50	89.83	90.18	90.99	92.52	89.60	89.83	89.83	90.05	90.18	90.18	89.95	90.42																				
CTLV-ASGV-2-HJY	MH144343	98.24	90.52	90.52	92.40	90.87	91.81	90.76	90.76	92.75	90.65	91.12	91.81	97.07	90.53	90.65	90.65	90.76	91.12	91.12	91.12	91.35	91.58																			
ASGV-Matsuco	LC084659	91.35	90.52	90.52	91.23	90.64	91.22	90.52	90.52	91.58	96.96	97.19	90.76	92.28	96.61	96.96	96.96	90.52	97.19	97.19	97.07	97.42	90.42	91.00																		
ASGV-FKSS2	LC143387	94.98	91.92	91.92	93.57	92.16	93.45	92.04	92.04	93.57	91.70	92.05	93.33	94.74	91.13	91.70	91.71	92.04	92.06	91.83	91.71	92.05	92.64	94.04	92.40																	
ASGV-N297	LC184610	94.50	91.81	91.81	93.22	92.04	93.33	91.92	91.92	93.45	91.23	91.35	93.21	94.50	90.77	91.23	91.23	91.92	91.35	91.35	91.35	91.58	92.99	93.92	91.93	98.24																
ASGV-Kiyomi	LC184611	92.16	96.37	96.37	95.43	96.37	98.59	96.02	96.02	92.28	90.40	91.11	99.06	91.92	90.52	90.40	90.40	96.02	91.11	90.99	90.76	91.34	91.22	91.81	90.76	93.33	93.45															
ASGV-Nagami	LC184612	94.73	90.52	90.52	92.04	90.40	91.81	90.76	90.76	92.74	90.17	90.99	91.69	95.08	90.64	90.17	90.17	90.76	90.99	91.46	91.34	91.34	92.51	93.68	90.05	93.45	93.56	91.92														
CTLV-L	D16681	95.33	91.57	91.57	92.99	92.04	93.09	91.92	91.92	93.45	91.00	91.58	92.98	95.09	90.66	91.00	91.01	91.92	91.59	91.13	91.24	91.58	92.28	94.62	91.70	98.24	97.89	92.98	93.80													
ASGV-Li-23	AB004063	95.21	91.81	91.81	93.22	92.28	93.33	92.16	92.16	93.45	91.00	91.58	93.21	95.21	91.00	91.00	91.00	92.16	91.58	91.35	91.58	91.82	92.52	94.62	91.93	98.01	98.13	93.21	93.80	99.76												
ASGV-P-209	NC001749	92.99	90.17	90.17	92.17	90.52	90.87	90.40	90.40	92.75	90.65	90.42	90.64	92.75	89.83	90.65	90.65	90.40	90.42	90.42	90.42	90.53	91.12	92.64	90.65	93.10	92.75	90.64	91.92	92.99	93.22											
ASGVp12	HE978837	92.87	90.87	90.87	92.17	90.64	91.34	90.52	90.52	91.82	90.53	90.30	91.22	92.40	89.95	90.53	90.53	90.52	90.30	90.77	90.53	90.77	89.95	92.40	90.77	93.57	92.87	91.46	91.11	93.34	93.34	91.82										
ASGV-AC	KX988001	93.22	90.52	90.52	91.82	90.29	91.22	90.17	90.17	92.05	90.77	90.53	91.11	92.99	90.18	90.77	90.77	90.17	90.53	91.00	90.77	91.00	90.18	92.99	91.00	94.04	93.57	91.34	91.81	93.80	93.80	91.93	98.71									
ASGV-HH	JN701424	93.10	90.99	90.99	92.40	91.11	92.16	90.87	90.87	92.40	91.23	91.23	91.57	93.10	90.88	91.23	91.23	90.87	91.23	91.47	91.23	91.70	91.23	92.52	91.12	94.39	93.80	91.92	92.28	94.39	94.39	92.75	92.40	93.22								
ASGV-241KP	D14995	92.99	90.17	90.17	92.17	90.52	90.87	90.40	90.40	92.75	90.65	90.42	90.64	92.75	89.83	90.65	90.65	90.40	90.42	90.42	90.42	90.53	91.12	92.64	90.65	93.10	92.75	90.64	91.92	92.99	93.22	100.00	91.82	91.93	92.75							
ASGV-kfp	KR106996	91.83	90.52	90.76	90.88	91.22	91.57	90.87	90.87	91.47	90.07	90.65	91.81	92.06	90.31	90.07	90.08	90.87	90.66	90.89	90.89	91.12	90.30	91.00	91.12	92.29	91.93	91.81	91.57	91.94	92.17	91.00	90.65	91.23	91.35	91.00						
ASGV-Ac	JX080201	93.34	90.99	90.99	92.28	91.22	92.39	91.11	91.11	91.93	90.77	91.35	91.92	93.69	91.00	90.77	90.77	91.11	91.35	91.35	91.58	91.82	90.65	92.75	91.35	94.62	94.27	92.16	92.39	93.92	94.15	91.58	92.87	92.87	92.75	91.58	92.75					
ASGV-CHN	JQ308181	92.40	90.52	90.52	91.23	90.29	90.87	90.40	90.40	91.82	90.88	90.65	90.40	92.17	90.42	90.88	90.88	90.40	90.65	91.12	90.65	91.12	91.00	91.23	90.88	93.45	92.87	90.64	91.92	93.45	93.45	92.28	91.82	92.28	95.79	92.28	90.77	92.05				
ASGV-YTG	KJ579253	93.80	91.69	91.69	93.34	91.92	93.09	91.57	91.57	93.92	91.93	91.82	92.39	94.04	91.47	91.93	91.93	91.57	91.82	92.75	91.82	92.28	92.40	93.45	92.28	94.62	94.04	92.63	93.33	94.39	94.39	93.69	93.69	93.80	96.49	93.69	92.17	93.45	94.97			
ASGV-HT	KU947036	91.13	89.94	89.94	90.65	89.70	90.76	89.59	89.59	89.95	88.78	89.95	90.05	91.36	89.26	88.78	88.68	89.59	89.84	89.38	89.73	89.95	88.66	91.12	89.95	90.89	90.53	90.17	90.64	91.24	91.35	88.55	90.30	90.88	90.07	88.55	90.01	90.07	89.25	91.35		
PBNLSV	AY596172	92.29	90.52	90.76	91.82	90.52	92.04	90.64	90.64	92.05	90.07	90.18	91.22	92.18	89.61	90.07	89.96	90.64	90.08	90.31	90.19	90.42	90.30	91.93	90.53	94.74	94.27	91.46	91.22	94.16	94.27	91.00	91.47	91.93	92.40	91.00	93.23	93.10	92.40	93.34	89.38	

CTLV: citrus tatter leaf virus; ASGV: apple stem grooving virus; PBNLSV: pear black necrotic leaf spot virus
