# Supplementary material for: Full genome characterization of 12 citrus tatter leaf virus isolates for the development of a detection assay
Source: PLoS One. 2019 Oct 17;14(10):e0223958. doi: 10.1371/journal.pone.0223958 (PMC6797102; doi:10.1371/journal.pone.0223958)
Supplement: S5 Table — (PDF) [file pone.0223958.s006.pdf]

**S5 Table. Nucleotide sequence identities (%) of movement protein (MP).**

| Isolate | GenBank | CTLV-IPPN122 | CTLV-TL100 | CTLV-TL101 | CTLV-TL102 | CTLV-TL103 | CTLV-TL104 | CTLV-TL110 | CTLV-TL111 | CTLV-TL112 | CTLV-TL113 | CTLV-TL114 | CTLV-TL115 | CTLV-MTH | CTLV-XHC | CTLV-Pk | CTLV-Ponkan8 | CTLV-ML | CTLV-Kumquat1 | CTLV-LCd-NA-1 | CTLV-Shatang Orange | CTLV-HJY | CTLV-ASGV-1-HJY | CTLV-ASGV-2-HJY | ASGV-Matsuco | ASGV-FKSS2 | ASGV-N297 | ASGV-Kiyomi | ASGV-Nagami | CTLV-L | ASGV-Li-23 | ASGV-P-209 | ASGVp12 | ASGV-AC | ASGV-HH | ASGV-241KP | ASGV-kfp | ASGV-Ac | ASGV-CHN | ASGV-YTG | ASGV-HT | PBNLSV |
|---|---|---|---|---|---|---|---|---|---|---|---|---|---|---|---|---|---|---|---|---|---|---|---|---|---|---|---|---|---|---|---|---|---|---|---|---|---|---|---|---|---|---|
| CTLV-IPPN122 | MH108986 | | | | | | | | | | | | | | | | | | | | | | | | | | | | | | | | | | | | | | | | | |
| CTLV-TL100 | MH108975 | 85.87 | | | | | | | | | | | | | | | | | | | | | | | | | | | | | | | | | | | | | | | | |
| CTLV-TL101 | MH108976 | 86.29 | 98.85 | | | | | | | | | | | | | | | | | | | | | | | | | | | | | | | | | | | | | | | |
| CTLV-TL102 | MH108977 | 86.18 | 98.44 | 98.96 | | | | | | | | | | | | | | | | | | | | | | | | | | | | | | | | | | | | | | |
| CTLV-TL103 | MH108978 | 86.08 | 98.54 | 98.85 | 98.44 | | | | | | | | | | | | | | | | | | | | | | | | | | | | | | | | | | | | | |
| CTLV-TL104 | MH108979 | 87.33 | 94.28 | 94.60 | 94.60 | 94.91 | | | | | | | | | | | | | | | | | | | | | | | | | | | | | | | | | | | | |
| CTLV-TL110 | MH108980 | 86.08 | 98.54 | 98.85 | 98.44 | 100.00 | 94.91 | | | | | | | | | | | | | | | | | | | | | | | | | | | | | | | | | | | |
| CTLV-TL111 | MH108981 | 86.08 | 98.54 | 98.85 | 98.44 | 100.00 | 94.91 | 100.00 | | | | | | | | | | | | | | | | | | | | | | | | | | | | | | | | | | |
| CTLV-TL112 | MH108982 | 85.56 | 88.47 | 88.05 | 87.64 | 88.16 | 86.91 | 88.16 | 88.16 | | | | | | | | | | | | | | | | | | | | | | | | | | | | | | | | | |
| CTLV-TL113 | MH108983 | 85.56 | 87.12 | 86.70 | 86.50 | 87.01 | 86.08 | 87.01 | 87.01 | 94.91 | | | | | | | | | | | | | | | | | | | | | | | | | | | | | | | | |
| CTLV-TL114 | MH108984 | 85.77 | 86.70 | 86.50 | 86.70 | 86.60 | 86.18 | 86.60 | 86.60 | 94.91 | 95.01 | | | | | | | | | | | | | | | | | | | | | | | | | | | | | | | |
| CTLV-TL115 | MH108985 | 86.39 | 93.66 | 93.76 | 93.97 | 94.49 | 96.88 | 94.49 | 94.49 | 86.18 | 85.56 | 86.29 | | | | | | | | | | | | | | | | | | | | | | | | | | | | | | |
| CTLV-MTH | KC588948 | 87.33 | 85.66 | 85.35 | 85.15 | 85.46 | 86.29 | 85.46 | 85.46 | 84.73 | 85.35 | 85.87 | 86.29 | | | | | | | | | | | | | | | | | | | | | | | | | | | | | |
| CTLV-XHC | KC588947 | 84.63 | 86.29 | 85.87 | 85.87 | 86.18 | 85.35 | 86.18 | 86.18 | 94.08 | 93.87 | 97.40 | 85.66 | 85.04 | | | | | | | | | | | | | | | | | | | | | | | | | | | | |
| CTLV-Pk | JX416228 | 85.56 | 87.12 | 86.70 | 86.50 | 87.01 | 86.08 | 87.01 | 87.01 | 94.91 | 100.00 | 95.01 | 85.56 | 85.35 | 93.87 | | | | | | | | | | | | | | | | | | | | | | | | | | | |
| CTLV-Ponkan8 | KY706358 | 85.56 | 87.12 | 86.70 | 86.50 | 87.01 | 86.08 | 87.01 | 87.01 | 94.91 | 100.00 | 95.01 | 85.56 | 85.35 | 93.87 | 100.00 | | | | | | | | | | | | | | | | | | | | | | | | | | |
| CTLV-ML | EU553489 | 86.08 | 98.54 | 98.85 | 98.44 | 100.00 | 94.91 | 100.00 | 100.00 | 88.16 | 87.01 | 86.60 | 94.49 | 85.46 | 86.18 | 87.01 | 87.01 | | | | | | | | | | | | | | | | | | | | | | | | | |
| CTLV-Kumquat1 | AY646511 | 85.77 | 86.70 | 86.50 | 86.70 | 86.60 | 86.18 | 86.60 | 86.60 | 94.91 | 95.01 | 100.00 | 86.29 | 85.87 | 97.40 | 95.01 | 95.01 | 86.60 | | | | | | | | | | | | | | | | | | | | | | | | |
| CTLV-LCd-NA-1 | FJ355920 | 85.87 | 88.47 | 88.05 | 87.64 | 88.16 | 86.91 | 88.16 | 88.16 | 99.58 | 95.32 | 95.11 | 86.18 | 85.04 | 94.49 | 95.32 | 95.32 | 88.16 | 95.11 | | | | | | | | | | | | | | | | | | | | | | | |
| CTLV-Shatang Orange | JQ765412 | 85.35 | 86.50 | 86.29 | 86.29 | 86.60 | 85.98 | 86.60 | 86.60 | 95.01 | 94.70 | 98.44 | 86.08 | 85.46 | 97.71 | 94.70 | 94.70 | 86.60 | 98.44 | 95.43 | | | | | | | | | | | | | | | | | | | | | | |
| CTLV-HJY | MH144341 | 85.46 | 86.91 | 86.50 | 86.50 | 86.81 | 86.39 | 86.81 | 86.81 | 94.80 | 94.60 | 97.61 | 86.50 | 85.77 | 97.09 | 94.60 | 94.60 | 86.81 | 97.61 | 95.22 | 97.71 | | | | | | | | | | | | | | | | | | | | | |
| CTLV-ASGV-1-HJY | MH144342 | 84.42 | 86.18 | 86.08 | 86.08 | 85.98 | 87.33 | 85.98 | 85.98 | 84.63 | 84.83 | 85.46 | 86.81 | 84.31 | 85.25 | 84.83 | 84.83 | 85.98 | 85.46 | 84.63 | 84.83 | 84.83 | | | | | | | | | | | | | | | | | | | | |
| CTLV-ASGV-2-HJY | MH144343 | 89.92 | 84.94 | 84.63 | 84.83 | 84.73 | 86.18 | 84.73 | 84.73 | 84.21 | 84.83 | 84.94 | 85.25 | 90.34 | 83.90 | 84.83 | 84.83 | 84.73 | 84.94 | 84.52 | 84.11 | 84.83 | 84.11 | | | | | | | | | | | | | | | | | | | |
| ASGV-Matsuco | LC084659 | 84.94 | 87.33 | 86.91 | 86.70 | 87.43 | 86.29 | 87.43 | 87.43 | 95.43 | 96.57 | 94.91 | 86.39 | 85.46 | 94.80 | 96.57 | 96.57 | 87.43 | 94.91 | 95.84 | 95.32 | 95.11 | 84.94 | 84.52 | | | | | | | | | | | | | | | | | | |
| ASGV-FKSS2 | LC143387 | 85.87 | 86.29 | 85.98 | 85.98 | 86.08 | 86.08 | 86.08 | 86.08 | 84.11 | 85.25 | 85.35 | 85.66 | 86.60 | 84.31 | 85.25 | 85.25 | 86.08 | 85.35 | 84.31 | 84.63 | 85.15 | 84.31 | 86.91 | 84.42 | | | | | | | | | | | | | | | | | |
| ASGV-N297 | LC184610 | 85.46 | 86.08 | 85.77 | 85.35 | 85.66 | 85.66 | 85.66 | 85.66 | 84.11 | 85.04 | 85.25 | 85.25 | 86.08 | 84.42 | 85.04 | 85.04 | 85.66 | 85.25 | 84.31 | 84.73 | 85.04 | 85.15 | 86.70 | 84.83 | 96.05 | | | | | | | | | | | | | | | | |
| ASGV-Kiyomi | LC184611 | 86.91 | 94.18 | 94.49 | 94.49 | 95.01 | 97.61 | 95.01 | 95.01 | 86.81 | 85.56 | 86.29 | 98.65 | 86.39 | 85.66 | 85.56 | 85.56 | 95.01 | 86.29 | 86.81 | 86.08 | 86.50 | 87.22 | 85.77 | 86.39 | 85.98 | 85.35 | | | | | | | | | | | | | | | |
| ASGV-Nagami | LC184612 | 87.12 | 85.98 | 85.66 | 85.46 | 86.18 | 86.60 | 86.18 | 86.18 | 85.87 | 85.25 | 85.35 | 86.39 | 92.52 | 84.31 | 85.25 | 85.25 | 86.18 | 85.35 | 85.98 | 85.15 | 85.25 | 84.73 | 91.38 | 85.25 | 86.18 | 86.29 | 86.70 | | | | | | | | | | | | | | |
| CTLV-L | D16681 | 84.63 | 84.83 | 84.73 | 84.52 | 84.83 | 84.11 | 84.83 | 84.83 | 83.07 | 85.04 | 84.11 | 84.21 | 84.63 | 83.28 | 85.04 | 85.04 | 84.83 | 84.11 | 83.28 | 83.59 | 83.90 | 83.80 | 85.66 | 83.80 | 95.84 | 94.80 | 84.52 | 84.52 | | | | | | | | | | | | | |
| ASGV-Li-23 | AB004063 | 84.94 | 85.25 | 85.15 | 84.94 | 85.04 | 84.31 | 85.04 | 85.04 | 83.28 | 85.25 | 84.31 | 84.21 | 84.94 | 83.48 | 85.25 | 85.25 | 85.04 | 84.31 | 83.48 | 83.80 | 84.11 | 84.11 | 85.98 | 84.00 | 96.26 | 95.43 | 84.52 | 84.63 | 98.75 | | | | | | | | | | | | |
| ASGV-P-209 | NC001749 | 87.43 | 85.25 | 84.52 | 84.73 | 84.83 | 85.87 | 84.83 | 84.83 | 85.66 | 85.35 | 86.08 | 85.46 | 91.58 | 85.35 | 85.35 | 85.35 | 84.83 | 86.08 | 85.98 | 85.56 | 86.08 | 84.00 | 90.23 | 85.66 | 86.91 | 86.70 | 85.56 | 92.93 | 84.73 | 85.15 | | | | | | | | | | | |
| ASGVp12 | HE978837 | 84.11 | 85.66 | 85.56 | 85.46 | 85.66 | 85.04 | 85.66 | 85.66 | 86.29 | 86.18 | 87.01 | 85.77 | 84.21 | 86.81 | 86.18 | 86.18 | 85.66 | 87.01 | 86.60 | 86.70 | 86.81 | 84.11 | 83.28 | 87.01 | 84.73 | 85.25 | 85.87 | 83.59 | 83.80 | 83.59 | 84.83 | | | | | | | | | | |
| ASGV-AC | KX988001 | 84.83 | 86.29 | 85.98 | 85.87 | 86.08 | 85.87 | 86.08 | 86.08 | 86.81 | 86.60 | 87.12 | 85.98 | 85.04 | 86.91 | 86.60 | 86.60 | 86.08 | 87.12 | 87.12 | 86.60 | 86.70 | 85.25 | 83.90 | 87.33 | 85.04 | 85.77 | 86.70 | 84.63 | 84.11 | 84.11 | 85.66 | 97.50 | | | | | | | | | |
| ASGV-HH | JN701424 | 84.52 | 84.42 | 84.00 | 84.21 | 83.80 | 84.94 | 83.80 | 83.80 | 83.90 | 85.35 | 84.52 | 84.63 | 84.83 | 83.17 | 85.35 | 85.35 | 83.80 | 84.52 | 84.21 | 84.11 | 84.42 | 83.80 | 85.35 | 84.42 | 88.57 | 88.99 | 84.73 | 85.87 | 87.64 | 87.43 | 86.50 | 83.80 | 84.11 | | | | | | | | |
| ASGV-241KP | D14995 | 87.43 | 85.25 | 84.52 | 84.73 | 84.83 | 85.87 | 84.83 | 84.83 | 85.66 | 85.35 | 86.08 | 85.46 | 91.58 | 85.35 | 85.35 | 85.35 | 84.83 | 86.08 | 85.98 | 85.56 | 86.08 | 84.00 | 90.23 | 85.66 | 86.91 | 86.70 | 85.56 | 92.93 | 84.73 | 85.15 | 100.00 | 84.83 | 85.66 | 86.50 | | | | | | | |
| ASGV-kfp | KR106996 | 84.52 | 84.42 | 84.52 | 84.42 | 84.63 | 85.46 | 84.63 | 84.63 | 83.17 | 84.21 | 83.90 | 85.66 | 84.42 | 83.17 | 84.21 | 84.21 | 84.63 | 83.90 | 83.38 | 83.48 | 84.00 | 84.83 | 84.00 | 84.83 | 83.69 | 84.63 | 85.35 | 83.69 | 83.48 | 83.69 | 83.07 | 85.66 | 86.60 | 83.90 | 83.07 | | | | | | |
| ASGV-Ac | JX080201 | 87.01 | 86.18 | 85.98 | 85.66 | 86.29 | 86.39 | 86.29 | 86.29 | 85.25 | 85.15 | 85.87 | 85.25 | 86.91 | 85.15 | 85.15 | 85.15 | 86.29 | 85.87 | 85.56 | 85.87 | 86.08 | 85.77 | 85.66 | 85.25 | 85.25 | 85.87 | 85.46 | 86.81 | 84.42 | 84.63 | 85.77 | 85.15 | 85.77 | 83.28 | 85.77 | 84.00 | | | | | |
| ASGV-CHN | JQ308181 | 84.21 | 83.69 | 83.48 | 83.48 | 83.07 | 84.52 | 83.07 | 83.07 | 82.24 | 84.11 | 83.07 | 84.31 | 84.31 | 81.82 | 84.11 | 84.11 | 83.07 | 83.07 | 82.55 | 82.65 | 83.17 | 82.86 | 84.83 | 83.07 | 87.53 | 87.64 | 84.42 | 85.15 | 86.08 | 85.77 | 86.18 | 83.07 | 83.38 | 94.49 | 86.18 | 82.65 | 84.11 | | | | |
| ASGV-YTG | KJ579253 | 86.18 | 86.60 | 86.50 | 86.39 | 86.18 | 87.01 | 86.18 | 86.18 | 86.18 | 86.60 | 86.70 | 86.08 | 85.56 | 86.08 | 86.60 | 86.60 | 86.18 | 86.70 | 86.39 | 86.18 | 85.87 | 85.56 | 86.08 | 86.08 | 86.70 | 86.70 | 86.60 | 85.87 | 85.56 | 85.98 | 85.87 | 86.29 | 86.81 | 85.98 | 85.87 | 85.77 | 86.91 | 84.11 | | | |
| ASGV-HT | KU947036 | 85.66 | 84.73 | 84.94 | 84.52 | 84.94 | 84.94 | 84.94 | 84.94 | 84.00 | 83.80 | 83.17 | 84.73 | 83.17 | 82.03 | 83.80 | 83.80 | 84.94 | 83.17 | 84.21 | 82.76 | 83.07 | 82.86 | 83.80 | 83.80 | 83.28 | 83.48 | 84.73 | 83.69 | 83.17 | 82.86 | 83.59 | 83.90 | 85.15 | 84.00 | 83.59 | 83.48 | 82.65 | 83.17 | 83.69 | | |
| PBNLSV | AY596172 | 84.83 | 84.73 | 84.94 | 84.83 | 84.73 | 85.04 | 84.73 | 84.73 | 84.31 | 84.42 | 85.35 | 85.25 | 84.00 | 84.63 | 84.42 | 84.42 | 84.73 | 85.35 | 84.31 | 84.83 | 84.94 | 84.00 | 84.73 | 83.90 | 83.28 | 84.42 | 85.35 | 84.11 | 83.07 | 83.28 | 84.73 | 87.43 | 87.64 | 82.86 | 84.73 | 85.56 | 84.63 | 82.34 | 86.39 | 83.17 | |

CTLV: citrus tatter leaf virus; ASGV: apple stem grooving virus; PBNLSV: pear black necrotic leaf spot virus
