# Supplementary material for: Full genome characterization of 12 citrus tatter leaf virus isolates for the development of a detection assay
Source: PLoS One. 2019 Oct 17;14(10):e0223958. doi: 10.1371/journal.pone.0223958 (PMC6797102; doi:10.1371/journal.pone.0223958)
Supplement: S6 Table — (PDF) [file pone.0223958.s007.pdf]

S6 Table. Nucleotide (below diagonal) and amino acid (above diagonal) sequences identites (%) of variable region I (VRI) of citrus tatter leaf virus and apple stem grooving virus isolated from citrus and citrus relati

Isolate	GenBank	CTLV-IPPN122	CTLV-TL100	CTLV-TL101	CTLV-TL102	CTLV-TL103	CTLV-TL104	CTLV-TL110	CTLV-TL111	CTLV-TL112	CTLV-TL113	CTLV-TL114	CTLV-TL115	CTLV-MTH	CTLV-XHC	CTLV-Pk	CTLV-Ponkan8	CTLV-ML	CTLV-Kumquat1	CTLV-LCd-NA-1	CTLV-Shatang Orange	CTLV-HJY	CTLV-ASGV-1-HJY	CTLV-ASGV-2-HJY	ASGV-Matsuco	ASGV-FKSS2	ASGV-N297	ASGV-Kiyomi	ASGV-Nagami
CTLV-IPPN122	MH108986		10.25	10.25	10.25	10.25	20.51	10.25	10.25	17.94	20.51	20.51	17.94	38.46	17.94	20.51	20.51	10.25	20.51	17.94	17.94	17.94	20.51	28.20	17.94	33.33	28.20	17.94	30.76
CTLV-TL100	MH108975	41.88		94.87	94.87	97.43	74.35	97.43	97.43	12.82	10.25	10.25	69.23	12.82	12.82	10.25	10.25	97.43	17.94	12.82	12.82	15.38	17.94	12.82	12.82	17.94	25.64	71.79	15.38
CTLV-TL101	MH108976	40.17	98.29		94.87	97.43	69.23	97.43	97.43	12.82	12.82	12.82	64.10	12.82	12.82	12.82	12.82	97.43	17.94	12.82	12.82	15.38	20.51	12.82	10.25	12.82	20.51	66.66	15.38
CTLV-TL102	MH108977	41.02	98.29	98.29		97.43	69.23	97.43	97.43	12.82	10.25	10.25	64.10	10.25	12.82	10.25	10.25	97.43	17.94	12.82	12.82	15.38	20.51	15.38	10.25	15.38	23.07	66.66	17.94
CTLV-TL103	MH108978	41.02	97.43	97.43	97.43		71.79	100.00	100.00	12.82	10.25	10.25	66.66	10.25	12.82	10.25	10.25	100.00	17.94	12.82	12.82	15.38	20.51	15.38	10.25	15.38	23.07	69.23	17.94
CTLV-TL104	MH108979	39.31	82.90	81.19	81.19	80.34		71.79	71.79	15.38	15.38	15.38	87.17	17.94	15.38	15.38	15.38	71.79	17.94	15.38	15.38	17.94	20.51	17.94	17.94	23.07	30.76	89.74	25.64
CTLV-TL110	MH108980	41.02	97.43	97.43	97.43	100.00	80.34		100.00	12.82	10.25	10.25	66.66	10.25	12.82	10.25	10.25	100.00	17.94	12.82	12.82	15.38	20.51	15.38	10.25	15.38	23.07	69.23	17.94
CTLV-TL111	MH108981	41.02	97.43	97.43	97.43	100.00	80.34	100.00		12.82	10.25	10.25	66.66	10.25	12.82	10.25	10.25	100.00	17.94	12.82	12.82	15.38	20.51	15.38	10.25	15.38	23.07	69.23	17.94
CTLV-TL112	MH108982	39.31	42.73	42.73	41.88	41.02	37.60	41.02	41.02		79.48	79.48	15.38	25.64	94.87	79.48	79.48	12.82	89.74	100.00	100.00	92.30	15.38	10.25	82.05	12.82	15.38	15.38	23.07
CTLV-TL113	MH108983	37.60	39.31	39.31	38.46	37.60	40.17	37.60	37.60	85.47		100.00	17.94	30.76	79.48	100.00	100.00	10.25	69.23	79.48	79.48	76.92	20.51	7.69	82.05	15.38	20.51	17.94	23.07
CTLV-TL114	MH108984	37.60	39.31	39.31	38.46	37.60	40.17	37.60	37.60	85.47	100.00		17.94	30.76	79.48	100.00	100.00	10.25	69.23	79.48	79.48	76.92	20.51	7.69	82.05	15.38	20.51	17.94	23.07
CTLV-TL115	MH108985	38.46	80.34	78.63	78.63	77.77	90.59	77.77	77.77	40.17	41.88	41.88		20.51	15.38	17.94	17.94	66.66	17.94	15.38	15.38	17.94	23.07	12.82	20.51	20.51	28.20	97.43	20.51
CTLV-MTH	KC588948	52.99	46.15	46.15	45.29	43.58	46.15	43.58	43.58	45.29	47.86	47.86	47.00		28.20	30.76	30.76	10.25	20.51	25.64	25.64	25.64	20.51	51.28	28.20	28.20	41.02	20.51	61.53
CTLV-XHC	KC588947	38.46	43.58	43.58	42.73	41.88	38.46	41.88	41.88	97.43	86.32	86.32	41.02	46.15		79.48	79.48	12.82	84.61	94.87	94.87	87.17	17.94	12.82	76.92	12.82	15.38	15.38	28.20
CTLV-Pk	JX416228	37.60	39.31	39.31	38.46	37.60	40.17	37.60	37.60	85.47	100.00	100.00	41.88	47.86	86.32		100.00	10.25	69.23	79.48	79.48	76.92	20.51	7.69	82.05	15.38	20.51	17.94	23.07
CTLV-Ponkan8	KY706358	37.60	39.31	39.31	38.46	37.60	40.17	37.60	37.60	85.47	100.00	100.00	41.88	47.86	86.32	100.00		10.25	69.23	79.48	79.48	76.92	20.51	7.69	82.05	15.38	20.51	17.94	23.07
CTLV-ML	EU553489	41.02	97.43	97.43	97.43	100.00	80.34	100.00	100.00	41.02	37.60	37.60	77.77	43.58	41.88	37.60	37.60		17.94	12.82	12.82	15.38	20.51	15.38	10.25	15.38	23.07	69.23	17.94
CTLV-Kumquat1	AY646511	41.02	43.58	43.58	42.73	41.88	40.17	41.88	41.88	93.16	82.05	82.05	42.73	44.44	92.30	82.05	82.05	41.88		89.74	89.74	82.05	15.38	7.69	74.35	15.38	17.94	17.94	17.94
CTLV-LCd-NA-1	FJ355920	39.31	42.73	42.73	41.88	41.02	37.60	41.02	41.02	100.00	85.47	85.47	40.17	45.29	97.43	85.47	85.47	41.02	93.16		100.00	92.30	15.38	10.25	82.05	12.82	15.38	15.38	23.07
CTLV-Shatang Orange	JQ765412	41.02	46.15	46.15	45.29	43.58	41.02	43.58	43.58	94.87	88.03	88.03	43.58	47.86	94.01	88.03	88.03	43.58	91.45	94.87		92.30	15.38	10.25	82.05	12.82	15.38	15.38	23.07
CTLV-HJY	MH144341	41.02	43.58	43.58	42.73	43.58	39.31	43.58	43.58	92.30	86.32	86.32	41.02	47.00	91.45	86.32	86.32	43.58	88.88	92.30	92.30		17.94	10.25	79.48	12.82	15.38	17.94	23.07
CTLV-ASGV-1-HJY	MH144342	41.88	41.02	41.88	41.88	41.02	39.31	41.02	41.02	37.60	37.60	37.60	41.02	40.17	37.60	37.60	37.60	41.02	41.02	37.60	42.73	41.88		12.82	15.38	15.38	17.94	23.07	23.07
CTLV-ASGV-2-HJY	MH144343	56.41	41.88	41.88	42.73	41.02	41.02	41.02	41.02	39.31	35.04	35.04	41.02	71.79	41.88	35.04	35.04	41.02	43.58	39.31	40.17	41.02	40.17		7.69	23.07	17.94	12.82	74.35
ASGV-Matsuco	LC084659	37.60	42.73	42.73	41.88	41.02	45.29	41.02	41.02	85.47	89.74	89.74	45.29	50.42	84.61	89.74	89.74	41.02	82.05	85.47	86.32	86.32	35.89	37.60		17.94	23.07	20.51	17.94
ASGV-FKSS2	LC143387	50.42	40.17	38.46	39.31	38.46	42.73	38.46	38.46	35.04	37.60	37.60	42.73	47.00	37.60	37.60	37.60	38.46	35.04	35.04	34.18	35.89	40.17	43.58	39.31		82.05	20.51	25.64
ASGV-N297	LC184610	48.71	45.29	43.58	44.44	43.58	47.86	43.58	43.58	38.46	42.73	42.73	47.86	53.84	41.02	42.73	42.73	43.58	38.46	38.46	37.60	39.31	40.17	43.58	42.73	90.59		28.20	28.20
ASGV-Kiyomi	LC184611	39.31	81.19	79.48	79.48	78.63	91.45	78.63	78.63	40.17	41.88	41.88	99.14	47.86	41.02	41.88	41.88	78.63	42.73	40.17	43.58	41.02	41.02	41.88	45.29	41.88	47.00		20.51
ASGV-Nagami	LC184612	54.70	43.58	43.58	44.44	42.73	45.29	42.73	42.73	44.44	43.58	43.58	45.29	75.21	47.00	43.58	43.58	42.73	47.00	44.44	45.29	46.15	41.02	86.32	44.44	45.29	48.71	46.15	

CTLV: citrus tatter leaf virus; ASGV: apple stem grooving virus
