# Supplementary material for: Full genome characterization of 12 citrus tatter leaf virus isolates for the development of a detection assay
Source: PLoS One. 2019 Oct 17;14(10):e0223958. doi: 10.1371/journal.pone.0223958 (PMC6797102; doi:10.1371/journal.pone.0223958)
Supplement: S7 Table — (PDF) [file pone.0223958.s008.pdf]

S7 Table. Nucleotide (below diagonal) and amino acid (above diagonal) sequences identities (%) of variable region II (VRII) of citrus tatter leaf virus and apple stem grooving virus isolated from citrus and citrus re

Isolate	GenBank	CTLV-IPPN122	CTLV-TL100	CTLV-TL101	CTLV-TL102	CTLV-TL103	CTLV-TL104	CTLV-TL110	CTLV-TL111	CTLV-TL112	CTLV-TL113	CTLV-TL114	CTLV-TL115	CTLV-MTH	CTLV-XHC	CTLV-Pk	CTLV-Ponkan8	CTLV-ML	CTLV-Kumquat1	CTLV-LCd-NA-1	CTLV-Shatang Orange	CTLV-HJY	CTLV-ASGV-1-HJY	CTLV-ASGV-2-HJY	ASGV-Matsuco	ASGV-FKSS2	ASGV-N297	ASGV-Kiyomi	ASGV-Nagami
CTLV-IPPN122	MH108986		58.74	59.44	59.79	59.79	62.58	59.79	59.79	57.69	56.64	57.69	60.13	62.93	53.84	56.64	56.64	59.79	57.69	57.69	56.64	56.29	56.99	71.67	54.19	57.34	56.64	61.18	63.28
CTLV-TL100	MH108975	84.26		96.50	95.10	95.45	82.51	95.45	95.45	66.78	63.28	62.58	80.41	58.39	61.53	63.28	63.28	95.45	62.58	66.78	62.93	62.93	61.53	56.64	63.98	60.13	59.09	81.46	59.79
CTLV-TL101	MH108976	84.73	98.71		97.20	96.85	83.56	96.85	96.85	65.73	62.23	62.23	80.76	57.69	60.13	62.23	62.23	96.85	62.23	65.73	62.58	61.88	61.18	56.29	62.93	59.44	58.74	82.51	59.44
CTLV-TL102	MH108977	84.61	98.25	98.83		95.45	83.56	95.45	95.45	64.33	61.53	62.93	81.46	56.99	60.13	61.53	61.53	95.45	62.93	64.33	62.58	61.88	61.18	56.99	62.23	59.44	57.34	82.51	58.74
CTLV-TL103	MH108978	84.61	98.48	98.83	98.36		84.61	100.00	100.00	66.43	63.63	62.93	82.51	58.39	61.53	63.63	63.63	100.00	62.93	66.43	63.98	63.28	60.83	56.99	65.03	60.13	58.74	84.26	61.53
CTLV-TL104	MH108979	86.01	93.70	94.05	94.05	94.52		84.61	84.61	61.18	58.74	59.79	90.90	60.13	57.34	58.74	58.74	84.61	59.79	61.18	60.13	60.13	64.68	60.13	60.13	59.79	57.69	92.65	61.53
CTLV-TL110	MH108980	84.61	98.48	98.83	98.36	100.00	94.52		100.00	66.43	63.63	62.93	82.51	58.39	61.53	63.63	63.63	100.00	62.93	66.43	63.98	63.28	60.83	56.99	65.03	60.13	58.74	84.26	61.53
CTLV-TL111	MH108981	84.61	98.48	98.83	98.36	100.00	94.52	100.00		66.43	63.63	62.93	82.51	58.39	61.53	63.63	63.63	100.00	62.93	66.43	63.98	63.28	60.83	56.99	65.03	60.13	58.74	84.26	61.53
CTLV-TL112	MH108982	84.38	87.52	87.06	86.59	87.29	85.66	87.29	87.29		87.41	86.36	60.13	55.59	84.61	87.41	87.41	66.43	86.36	100.00	88.11	86.71	55.94	55.24	89.16	53.49	52.44	61.53	59.44
CTLV-TL113	MH108983	84.03	86.24	85.78	85.54	86.24	84.96	86.24	86.24	95.22		84.96	58.39	55.59	82.16	100.00	100.00	63.63	84.96	87.41	85.31	83.56	55.59	55.24	90.55	55.59	53.84	57.69	56.64
CTLV-TL114	MH108984	84.14	85.66	85.43	85.66	85.66	84.96	85.66	85.66	94.87	94.75		61.18	56.99	93.00	84.96	84.96	62.93	100.00	86.36	96.85	92.65	57.34	55.24	84.61	56.29	55.24	60.48	58.04
CTLV-TL115	MH108985	84.96	93.00	93.12	93.35	93.82	96.73	93.82	93.82	85.08	84.61	85.31		60.83	59.79	58.39	58.39	82.51	61.18	60.13	61.88	61.88	63.63	57.69	61.18	59.44	57.34	96.15	61.88
CTLV-MTH	KC588948	85.89	84.14	83.79	83.56	84.03	84.96	84.03	84.03	83.56	83.91	84.38	84.96		55.94	55.59	55.59	58.39	56.99	55.59	56.99	56.64	55.94	70.27	55.94	59.79	57.69	60.48	79.02
CTLV-XHC	KC588947	82.86	85.19	84.73	84.73	85.19	84.03	85.19	85.19	94.17	93.47	97.31	84.61	83.44		82.16	82.16	61.53	93.00	84.61	94.75	91.95	58.04	53.14	84.61	53.49	53.14	59.09	55.94
CTLV-Pk	JX416228	84.03	86.24	85.78	85.54	86.24	84.96	86.24	86.24	95.22	100.00	94.75	84.61	83.91	93.47		100.00	63.63	84.96	87.41	85.31	83.56	55.59	55.24	90.55	55.59	53.84	57.69	56.64
CTLV-Ponkan8	KY706358	84.03	86.24	85.78	85.54	86.24	84.96	86.24	86.24	95.22	100.00	94.75	84.61	83.91	93.47	100.00		63.63	84.96	87.41	85.31	83.56	55.59	55.24	90.55	55.59	53.84	57.69	56.64
CTLV-ML	EU553489	84.61	98.48	98.83	98.36	100.00	94.52	100.00	100.00	87.29	86.24	85.66	93.82	84.03	85.19	86.24	86.24		62.93	66.43	63.98	63.28	60.83	56.99	65.03	60.13	58.74	84.26	61.53
CTLV-Kumquat1	AY646511	84.14	85.66	85.43	85.66	85.66	84.96	85.66	85.66	94.87	94.75	100.00	85.31	84.38	97.31	94.75	94.75	85.66		86.36	96.85	92.65	57.34	55.24	84.61	56.29	55.24	60.48	58.04
CTLV-LCd-NA-1	FJ355920	84.38	87.52	87.06	86.59	87.29	85.66	87.29	87.29	100.00	95.22	94.87	85.08	83.56	94.17	95.22	95.22	87.29	94.87		88.11	86.71	55.94	55.24	89.16	53.49	52.44	61.53	59.44
CTLV-Shatang Orange	JQ765412	83.79	85.54	85.31	85.31	85.78	84.84	85.78	85.78	95.33	94.52	98.60	85.19	84.03	97.78	94.52	94.52	85.78	98.60	95.33		94.40	57.34	53.14	87.06	55.24	54.54	61.18	58.04
CTLV-HJY	MH144341	83.68	85.78	85.31	85.31	85.78	85.08	85.78	85.78	94.87	94.17	97.43	85.43	84.14	96.85	94.17	94.17	85.78	97.43	94.87	97.66		55.94	54.19	85.31	55.59	54.19	61.18	57.69
CTLV-ASGV-1-HJY	MH144342	83.33	85.66	85.54	85.54	85.54	87.06	85.54	85.54	83.44	83.68	84.26	86.48	83.33	84.26	83.68	83.68	85.54	84.26	83.44	83.91	83.68		55.94	56.64	56.29	57.69	64.33	56.99
CTLV-ASGV-2-HJY	MH144343	88.69	83.21	82.86	83.10	83.10	84.73	83.10	83.10	82.86	83.21	83.21	83.68	89.27	82.05	83.21	83.21	83.10	83.21	82.86	82.40	82.98	82.98		54.19	60.48	59.44	58.74	74.82
ASGV-Matsuco	LC084659	83.21	86.36	85.89	85.66	86.59	85.31	86.59	86.59	95.92	96.73	94.75	85.43	83.91	94.40	96.73	96.73	86.59	94.75	95.92	95.22	94.87	83.91	82.75		53.14	53.49	60.48	57.34
ASGV-FKSS2	LC143387	84.14	85.08	84.73	84.73	84.96	84.84	84.96	84.96	82.75	83.79	83.79	84.49	85.08	82.63	83.79	83.79	84.96	83.79	82.75	83.10	83.44	83.56	85.31	82.86		87.76	59.79	58.39
ASGV-N297	LC184610	83.68	84.84	84.49	84.03	84.49	84.38	84.49	84.49	82.75	83.56	83.68	84.03	84.49	82.75	83.56	83.56	84.49	83.68	82.75	83.21	83.33	84.49	85.08	83.33	95.57		56.99	59.09
ASGV-Kiyomi	LC184611	85.43	93.47	93.82	93.82	94.52	97.43	94.52	94.52	85.66	84.49	85.19	98.60	84.96	84.49	84.49	84.49	94.52	85.19	85.66	85.08	85.31	86.82	84.14	85.31	84.73	84.03		62.23
ASGV-Nagami	LC184612	86.13	84.61	84.26	84.03	84.96	85.43	84.96	84.96	84.49	83.91	84.14	85.19	92.30	82.98	83.91	83.91	84.96	84.14	84.49	84.03	83.91	83.44	90.90	84.03	84.96	85.08	85.43	

CTLV: citrus tatter leaf virus; ASGV: apple stem grooving virus
