# Supplementary material for: Full genome characterization of 12 citrus tatter leaf virus isolates for the development of a detection assay
Source: PLoS One. 2019 Oct 17;14(10):e0223958. doi: 10.1371/journal.pone.0223958 (PMC6797102; doi:10.1371/journal.pone.0223958)
Supplement: S8 Table — (PDF) [file pone.0223958.s009.pdf]

S8 Table. Nucleotide (below diagonal) and amino acid (above diagonal) sequence identities (%) of polyprotein (PP).

Isolate	Genbank Accession No.	CTLV-IPPN122	CTLV-TL100	CTLV-TL101	CTLV-TL102	CTLV-TL103	CTLV-TL104	CTLV-TL110	CTLV-TL111	CTLV-TL112	CTLV-TL113	CTLV-TL114	CTLV-TL115	CTLV-MTH	CTLV-XHC	CTLV-Pk	CTLV-Ponkan8	CTLV-ML	CTLV-Kumquat1	CTLV-LCd-NA-1	CTLV-Shatang Orange	CTLV-HJY	CTLV-ASGV-1-HJY	CTLV-ASGV-2-HJY	CTLV-L	ASGV-Li-23	ASGV-P-209	ASGVp12	ASGV-AC	ASGV-HH	ASGV-241KP	ASGV-Matsuco	ASGV-FKSS2	ASGV-N297	ASGV-Kiyomi	ASGV-Nagami	ASGV-kfp	ASGV-Ac	ASGV-CHN	ASGV-YTG	ASGV-HT	PBNLSV
CTLV-IPPN122	MH108986		86.24	86.34	86.29	86.13	86.83	86.13	86.13	86.02	86.13	86.34	86.18	89.40	84.95	86.08	86.08	86.13	85.81	85.97	85.65	85.76	86.88	90.73	86.08	85.76	89.66	82.55	85.59	87.68	89.66	85.81	87.52	87.04	86.24	89.77	83.46	86.67	86.88	87.09	84.47	84.20
CTLV-TL100	MH108975	80.62		99.25	98.92	98.66	94.27	98.66	98.66	87.04	86.34	86.40	93.73	86.24	85.81	86.40	86.40	98.66	86.45	86.99	86.24	86.61	87.20	86.18	85.49	85.27	86.40	83.62	86.40	86.40	86.40	86.72	86.61	86.93	94.00	86.99	84.00	86.13	85.43	85.86	83.88	84.36
CTLV-TL101	MH108976	80.71	98.89		99.25	98.98	94.43	98.98	98.98	86.88	86.34	86.50	93.79	86.13	85.70	86.40	86.40	98.98	86.50	86.83	86.24	86.45	87.20	86.13	85.38	85.11	86.18	83.57	86.24	86.34	86.18	86.61	86.40	86.77	94.16	86.93	83.95	86.02	85.38	85.70	83.88	84.20
CTLV-TL102	MH108977	80.67	98.60	98.85		98.55	94.21	98.55	98.55	86.56	85.97	86.34	93.68	85.86	85.49	86.02	86.02	98.55	86.40	86.50	86.08	86.34	87.20	86.29	85.33	85.06	86.13	83.46	86.13	86.29	86.13	86.29	86.34	86.50	93.95	86.88	83.79	85.86	85.33	85.70	83.72	84.15
CTLV-TL103	MH108978	80.67	98.41	98.62	98.26		94.27	100.00	100.00	86.72	86.24	86.29	93.73	85.97	85.65	86.29	86.29	100.00	86.34	86.67	86.18	86.40	86.77	86.08	85.38	85.01	86.24	83.41	86.08	86.02	86.24	86.67	86.34	86.56	94.11	87.09	83.68	85.92	85.06	85.49	83.77	84.04
CTLV-TL104	MH108979	80.38	92.02	92.32	92.09	91.97		94.27	94.27	86.18	86.24	86.24	97.05	86.61	85.27	86.29	86.29	94.27	86.13	86.13	85.92	86.24	87.68	86.67	85.22	84.74	86.61	83.57	86.24	86.08	86.61	86.29	86.83	86.72	97.37	87.36	84.16	86.13	86.18	85.81	83.99	84.10
CTLV-TL110	MH108980	80.67	98.41	98.62	98.26	100.00	91.97		100.00	86.72	86.24	86.29	93.73	85.97	85.65	86.29	86.29	100.00	86.34	86.67	86.18	86.40	86.77	86.08	85.38	85.01	86.24	83.41	86.08	86.02	86.24	86.67	86.34	86.56	94.11	87.09	83.68	85.92	85.06	85.49	83.77	84.04
CTLV-TL111	MH108981	80.67	98.41	98.62	98.26	100.00	91.97	100.00		86.72	86.24	86.29	93.73	85.97	85.65	86.29	86.29	100.00	86.34	86.67	86.18	86.40	86.77	86.08	85.38	85.01	86.24	83.41	86.08	86.02	86.24	86.67	86.34	86.56	94.11	87.09	83.68	85.92	85.06	85.49	83.77	84.04
CTLV-TL112	MH108982	79.69	80.65	80.56	80.44	80.56	80.29	80.56	80.56		95.93	95.44	86.13	86.18	95.61	95.77	95.77	86.72	96.14	99.94	96.57	96.46	85.65	86.13	85.22	84.85	86.93	84.64	87.36	85.65	86.93	96.09	86.02	85.92	86.18	87.15	83.73	86.29	84.79	86.18	84.26	84.42
CTLV-TL113	MH108983	79.55	80.76	80.69	80.62	80.65	80.69	80.65	80.65	94.09		96.84	86.13	86.50	94.59	99.83	99.83	86.24	95.02	95.87	95.34	95.23	85.86	86.29	86.13	85.65	86.83	84.75	87.31	86.08	86.83	96.52	86.40	86.34	85.86	86.93	84.38	85.92	85.54	86.29	84.10	84.36
CTLV-TL114	MH108984	79.90	80.65	80.65	80.54	80.54	80.96	80.54	80.54	92.80	96.25		86.40	86.77	96.25	96.68	96.68	86.29	97.43	95.39	97.00	96.41	86.34	86.45	85.70	85.22	87.41	84.97	87.47	85.81	87.41	95.28	86.45	86.50	86.13	87.20	84.27	86.34	85.17	86.29	83.72	84.95
CTLV-TL115	MH108985	80.13	91.59	91.70	91.55	91.39	95.37	91.39	91.39	80.29	80.71	81.08		86.67	85.76	86.18	86.18	93.73	86.45	86.08	86.29	86.56	87.47	86.08	85.43	84.79	86.56	83.78	86.29	86.02	86.56	86.50	86.61	86.56	98.87	87.31	84.32	85.43	86.02	85.49	83.77	84.10
CTLV-MTH	KC588948	85.01	80.65	80.58	80.44	80.53	80.88	80.53	80.53	80.58	80.54	81.06	80.79		85.76	86.56	86.56	85.97	86.13	86.13	86.24	86.50	86.77	92.29	86.72	86.29	93.68	83.19	86.02	88.54	93.68	86.50	88.22	88.11	86.50	94.11	84.11	86.72	88.75	87.63	83.94	83.88
CTLV-XHC	KC588947	79.24	80.04	79.99	79.99	80.06	79.99	80.06	80.06	94.84	93.20	94.27	80.28	80.31		94.53	94.53	85.65	97.21	95.55	97.75	97.26	85.49	85.22	84.74	84.47	86.40	84.27	86.83	84.85	86.40	94.80	85.33	85.43	85.49	86.08	83.30	85.38	83.88	85.43	82.76	84.31
CTLV-Pk	JX416228	79.47	80.63	80.56	80.49	80.53	80.63	80.53	80.53	93.54	99.37	96.09	80.69	80.62	93.29		100.00	86.29	94.96	95.71	95.28	95.18	85.81	86.18	85.97	85.49	86.77	84.59	87.15	85.97	86.77	96.46	86.24	86.18	85.92	86.88	84.38	85.81	85.59	86.24	84.20	84.42
CTLV-Ponkan8	KY706358	79.47	80.63	80.56	80.49	80.53	80.63	80.53	80.53	93.54	99.37	96.09	80.69	80.62	93.29	100.00		86.29	94.96	95.71	95.28	95.18	85.81	86.18	85.97	85.49	86.77	84.59	87.15	85.97	86.77	96.46	86.24	86.18	85.92	86.88	84.38	85.81	85.59	86.24	84.20	84.42
CTLV-ML	EU553489	80.67	98.41	98.62	98.26	100.00	91.97	100.00	100.00	80.56	80.65	80.54	91.39	80.53	80.06	80.53	80.53		86.34	86.67	86.18	86.40	86.77	86.08	85.38	85.01	86.24	83.41	86.08	86.02	86.24	86.67	86.34	86.56	94.11	87.09	83.68	85.92	85.06	85.49	83.77	84.04
CTLV-Kumquat1	AY646511	79.74	80.53	80.51	80.53	80.49	80.47	80.49	80.49	94.71	93.41	94.84	80.72	80.81	97.50	93.50	93.50	80.49		96.09	98.28	97.37	85.54	85.81	85.59	85.22	86.99	84.54	87.15	85.43	86.99	94.91	86.24	86.02	86.18	86.56	83.73	86.13	84.58	86.02	83.29	84.52
CTLV-LCd-NA-1	FJ355920	79.62	80.65	80.56	80.44	80.56	80.26	80.56	80.56	99.85	94.16	92.80	80.29	80.44	94.84	93.54	93.54	80.56	94.71		96.52	96.41	85.59	86.08	85.17	84.79	86.88	84.59	87.31	85.59	86.88	96.03	85.97	85.86	86.13	87.09	83.68	86.24	84.74	86.13	84.20	84.36
CTLV-Shatang Orange	JQ765412	79.81	80.29	80.24	80.26	80.33	80.19	80.33	80.33	94.98	93.70	94.68	80.47	80.74	97.93	93.71	93.71	80.33	97.91	94.98		98.01	85.54	85.54	85.43	85.01	86.93	84.54	86.99	85.49	86.93	95.55	86.02	85.92	86.02	86.67	83.68	86.34	84.63	85.92	83.40	84.58
CTLV-HJY	MH144341	79.67	80.53	80.40	80.47	80.54	80.28	80.54	80.54	94.45	93.30	94.11	80.51	80.67	96.98	93.39	93.39	80.54	96.93	94.48	97.32		85.70	85.86	85.33	84.95	87.15	84.59	87.04	85.49	87.15	95.50	86.18	86.02	86.29	86.88	84.27	86.56	84.90	85.81	83.77	84.79
CTLV-ASGV-1-HJY	MH144342	80.74	80.58	80.53	80.51	80.44	81.42	80.44	80.44	79.37	79.47	79.87	81.12	80.67	79.28	79.49	79.49	80.44	79.37	79.37	79.42	79.49		87.04	85.43	85.11	86.99	83.68	87.04	86.02	86.99	85.76	86.29	86.67	87.52	87.36	85.02	86.93	85.81	86.24	84.20	85.01
CTLV-ASGV-2-HJY	MH144343	85.47	79.99	79.80	79.94	79.80	80.31	79.80	79.80	80.17	80.19	80.49	79.96	89.82	79.69	80.19	80.19	79.80	80.21	80.03	79.94	80.08	80.83		86.77	86.50	93.68	83.09	86.18	89.29	93.68	86.02	88.49	88.27	86.13	94.27	84.11	86.50	89.02	88.06	84.15	84.90
CTLV-L	D16681	81.56	79.65	79.58	79.63	79.69	79.49	79.69	79.69	79.69	79.96	79.90	79.65	81.56	79.30	80.04	80.04	79.69	79.76	79.65	79.53	79.46	80.35	81.26		97.80	87.25	82.66	85.43	88.06	87.25	85.27	95.07	94.75	85.38	86.72	82.92	84.79	87.15	85.38	83.56	82.76
ASGV-Li-23	AB004063	81.53	79.55	79.47	79.53	79.55	79.38	79.55	79.55	79.71	79.92	79.85	79.40	81.37	79.24	80.01	80.01	79.55	79.65	79.67	79.49	79.44	80.22	81.33	98.30		86.93	82.12	84.95	87.74	86.93	84.79	94.70	94.59	84.79	86.24	82.66	84.47	86.56	85.17	83.08	82.49
ASGV-P-209	NC001749	84.92	80.49	80.22	80.31	80.22	80.33	80.22	80.22	80.33	80.17	80.54	80.46	90.75	79.92	80.26	80.26	80.22	80.47	80.22	80.24	80.46	81.08	92.27	81.37	81.31		84.05	86.88	89.93	100.00	86.99	88.97	88.75	86.40	94.86	83.89	86.67	89.82	87.90	84.31	84.74
ASGVp12	HE978837	79.56	80.05	80.08	79.85	79.96	80.01	79.96	79.96	80.30	80.30	80.56	79.90	79.71	80.08	80.15	80.15	79.96	80.15	80.22	80.12	80.19	80.44	79.13	78.81	78.86	80.12		95.06	83.52	84.05	84.75	83.41	83.89	83.73	83.41	81.42	83.41	83.14	83.62	81.91	83.09
ASGV-AC	KX988001	80.28	80.54	80.54	80.38	80.38	80.56	80.38	80.38	80.69	80.54	80.56	80.44	80.28	80.44	80.40	80.40	80.38	80.40	80.65	80.37	80.29	81.44	80.24	79.71	79.69	81.13	97.08		86.34	86.88	87.25	86.08	86.77	86.50	86.24	84.70	86.18	85.65	86.56	85.01	85.65
ASGV-HH	JN701424	83.17	79.71	79.71	79.56	79.53	79.74	79.53	79.53	79.96	80.51	80.29	79.46	85.40	79.44	80.46	80.46	79.53	79.96	79.83	80.08	79.88	80.33	86.34	82.44	82.42	86.18	79.33	80.04		89.93	85.54	89.29	89.29	86.08	89.56	83.68	85.22	93.09	87.15	83.83	83.51
ASGV-241KP	D14995	84.92	80.49	80.22	80.31	80.22	80.33	80.22	80.22	80.33	80.17	80.54	80.46	90.75	79.92	80.26	80.26	80.22	80.47	80.22	80.24	80.46	81.08	92.27	81.37	81.31	100.00	80.12	81.13	86.18		86.99	88.97	88.75	86.40	94.86	83.89	86.67	89.82	87.90	84.31	84.74
ASGV-Matsuco	LC084659	79.46	80.44	80.38	80.29	80.44	80.24	80.44	80.44	93.77	94.75	93.36	80.51	80.97	93.68	94.84	94.84	80.44	93.62	93.77	94.02	93.66	79.19	79.99	79.69	79.65	80.35	80.03	80.17	80.10	80.35		85.86	86.13	86.24	86.83	84.16	85.86	84.90	86.08	83.83	84.36
ASGV-FKSS2	LC143387	82.17	80.12	79.94	80.06	79.97	80.31	79.97	79.97	80.06	80.13	80.26	80.21	82.40	79.46	80.12	80.12	79.97	79.94	79.99	79.74	79.65	80.62	82.19	94.36	94.18	81.87	79.51	80.31	83.35	81.87	80.04		96.14	86.67	88.27	83.57	85.92	88.27	86.72	84.10	83.67
ASGV-N297	LC184610	81.58	80.19	79.87	79.99	79.94	80.06	79.94	79.94	79.88	79.96	80.13	79.88	82.36	79.49	80.04	80.04	79.94	79.96	79.81	79.78	79.55	80.65	82.01	93.66	93.39	81.72	79.56	80.44	82.90	81.72	80.04	94.78		86.40	88.49	84.22	86.02	88.22	86.50	84.04	84.15
ASGV-Kiyomi	LC184611	80.24	91.86	92.04	91.86	91.73	95.52	91.73	91.73	80.28	80.58	80.92	98.64	80.85	80.28	80.60	80.60	91.73	80.69	80.24	80.44	80.44	81.17	80.03	79.65	79.40	80.51	80.06	80.74	79.60	80.51	80.47	80.24	79.99		87.15	84.00	85.38	85.97	85.54	83.45	83.94
ASGV-Nagami	LC184612	85.26	81.01	80.85	80.88	80.92	80.97	80.92	80.92	80.99	80.88	80.92	80.67	90.86	80.38	80.97	80.97	80.92	80.78	80.88	80.87	80.85	80.97	92.93	81.26	81.28	93.12	79.88	80.71	86.24	93.12	80.99	82.13	82.11	80.78		84.22	87.20	89.23	87.90	84.26	84.47
ASGV-kfp	KR106996	77.86	77.83	77.88	77.72	77.77	77.90	77.77	77.77	78.33	78.35	78.53	77.84	78.08	78.26	78.56	78.56	77.77	78.38	78.29	78.40	78.58	78.72	78.19	77.63	77.58	77.88	77.45	78.31	77.83	77.88	78.56	77.99	78.15	77.74	78.38		83.89	83.19	83.79	82.76	84.32
ASGV-Ac	JX080201	80.44	80.03	79.85	79.78	79.96	80.29	79.96	79.96	80.96	80.88	81.15	80.03	79.92	80.67	80.81	80.81	79.96	80.78	80.99	80.83	81.01	80.74	80.15	79.65	79.47	80.31	80.08	80.60	79.51	80.31	80.74	80.21	80.13	79.92	80.53	77.34		85.17	86.13	82.76	84.47
ASGV-CHN	JQ308181	82.31	80.10	80.03	80.12	80.10	80.38	80.10	80.10	79.94	80.10	79.90	79.81	84.74	79.35	80.10	80.10	80.10	79.67	79.85	79.83	79.69	79.97	85.09	81.47	81.37	85.36	79.13	79.80	89.70	85.36	79.90	81.94	81.81	79.92	84.99	77.77	79.81		85.81	83.56	83.19
ASGV-YTG	KJ579253	83.01	80.49	80.44	80.46	80.40	79.92	80.40	80.40	80.42	80.79	80.71	79.76	85.42	80.31	80.88	80.88	80.40	80.88	80.42	80.71	80.60	81.01	85.08	81.22	80.92	85.65	80.24	80.76	82.60	85.65	80.94	81.54	81.19	79.81	86.08	78.10	80.22	81.35		84.52	85.01
ASGV-HT	KU947036	77.46	78.24	78.12	78.15	78.15	77.71	78.15	78.15	77.64	77.64	78.06	77.85	77.83	77.37	77.73	77.73	78.15	77.62	77.65	77.69	77.56	77.26	77.23	77.03	76.90	77.62	77.16	77.87	77.67	77.62	77.56	77.42	77.30	77.76	77.65	77.52	77.26	77.28	79.26		83.02
PBNLSV	AY596172	78.10	77.78	77.80	77.81	77.58	78.21	77.58	77.58	77.15	77.15	77.62	77.65	77.87	77.05	77.26	77.26	77.58	77.21	77.10	77.15	77.62	77.55	77.90	78.24	78.24	77.94	78.01	78.17	77.80	77.94	77.58	78.37	78.44	77.46	77.96	77.00	78.40	77.98	78.21	76.71	

CTLV: citrus tatter leaf virus; ASGV: apple stem grooving virus; PBNLSV: pear black necrotic leaf spot virus
