# Supplementary material for: Full genome characterization of 12 citrus tatter leaf virus isolates for the development of a detection assay
Source: PLoS One. 2019 Oct 17;14(10):e0223958. doi: 10.1371/journal.pone.0223958 (PMC6797102; doi:10.1371/journal.pone.0223958)
Supplement: S9 Table — (PDF) [file pone.0223958.s010.pdf]

S9 Table. Nucleotide (below diagonal) and amino acid (above diagonal) sequence identities (%) of coat protein (CP).

Isolate	Genbank Accession No.	CTLV-IPPN122	CTLV-TL100	CTLV-TL101	CTLV-TL102	CTLV-TL103	CTLV-TL104	CTLV-TL110	CTLV-TL111	CTLV-TL112	CTLV-TL113	CTLV-TL114	CTLV-TL115	CTLV-MTH	CTLV-XHC	CTLV-Pk	CTLV-Ponkan8	CTLV-ML	CTLV-Kumquat1	CTLV-LCd-NA-1	CTLV-Shatang Orange	CTLV-HJY	CTLV-ASGV-1-HJY	CTLV-ASGV-2-HJY	CTLV-L	ASGV-Li-23	ASGV-P-209	ASGVp12	ASGV-AC	ASGV-HH	ASGV-241KP	ASGV-Matsuco	ASGV-FKSS2	ASGV-N297	ASGV-Kiyomi	ASGV-Nagami	ASGV-kfp	ASGV-Ac	ASGV-CHN	ASGV-YTG	ASGV-HT	PBNLSV
CTLV-IPPN122	MH108986		94.53	94.95	96.21	94.95	95.37	94.95	94.95	94.95	94.95	94.95	94.95	98.73	94.11	94.95	94.95	94.95	94.95	96.21	94.53	95.37	93.69	100.00	97.47	97.47	96.63	97.47	97.05	97.05	96.63	94.53	97.05	96.63	95.37	96.21	94.53	97.05	96.63	97.47	95.37	94.95
CTLV-TL100	MH108975	91.31		99.57	97.05	99.57	97.89	99.57	99.57	95.37	92.01	93.69	97.05	94.53	92.43	92.01	92.01	99.57	93.69	94.11	92.85	93.69	93.27	94.53	95.79	95.79	94.11	95.37	95.37	95.37	94.11	93.69	95.37	95.79	97.89	94.11	94.53	94.11	95.37	96.63	94.53	94.95
CTLV-TL101	MH108976	91.59	99.71		97.47	100.00	98.31	100.00	100.00	95.79	92.01	94.11	97.47	94.95	92.85	92.01	92.01	100.00	94.11	94.53	93.27	94.11	93.69	94.95	96.21	96.21	94.53	95.79	95.79	95.79	94.53	93.69	95.79	96.21	98.31	94.53	94.95	94.53	94.95	97.05	94.95	95.37
CTLV-TL102	MH108977	93.27	96.63	96.91		97.47	97.05	97.47	97.47	98.31	93.27	95.37	97.05	95.79	94.11	93.27	93.27	97.47	95.37	95.79	94.53	95.37	94.11	96.21	97.05	97.05	95.79	97.05	96.63	96.63	95.79	94.95	97.05	96.63	97.05	96.21	94.95	95.37	96.21	97.89	95.79	96.21
CTLV-TL103	MH108978	91.59	99.15	99.43	96.35		98.31	100.00	100.00	95.79	92.01	94.11	97.47	94.95	92.85	92.01	92.01	100.00	94.11	94.53	93.27	94.11	93.69	94.95	96.21	96.21	94.53	95.79	95.79	95.79	94.53	93.69	95.79	96.21	98.31	94.53	94.95	94.53	94.95	97.05	94.95	95.37
CTLV-TL104	MH108979	91.87	96.49	96.77	95.37	96.21		98.31	98.31	95.37	92.01	94.11	99.15	94.53	92.85	92.01	92.01	98.31	94.11	94.95	93.27	94.11	94.11	95.37	96.63	96.63	94.95	96.21	96.21	96.21	94.95	93.69	96.21	96.63	100.00	95.79	95.37	94.95	95.37	97.47	95.37	95.79
CTLV-TL110	MH108980	91.59	99.15	99.43	96.35	100.00	96.21		100.00	95.79	92.01	94.11	97.47	94.95	92.85	92.01	92.01	100.00	94.11	94.53	93.27	94.11	93.69	94.95	96.21	96.21	94.53	95.79	95.79	95.79	94.53	93.69	95.79	96.21	98.31	94.53	94.95	94.53	94.95	97.05	94.95	95.37
CTLV-TL111	MH108981	91.59	99.15	99.43	96.35	100.00	96.21	100.00		95.79	92.01	94.11	97.47	94.95	92.85	92.01	92.01	100.00	94.11	94.53	93.27	94.11	93.69	94.95	96.21	96.21	94.53	95.79	95.79	95.79	94.53	93.69	95.79	96.21	98.31	94.53	94.95	94.53	94.95	97.05	94.95	95.37
CTLV-TL112	MH108982	92.43	91.59	91.87	94.67	91.59	92.43	91.59	91.59		92.01	94.11	95.37	94.53	92.85	92.01	92.01	95.79	94.11	94.53	93.27	94.11	93.27	94.95	95.79	95.79	94.53	95.79	95.37	95.37	94.53	93.69	95.79	95.37	95.37	94.95	93.27	94.11	94.95	96.21	94.11	94.53
CTLV-TL113	MH108983	90.19	90.05	90.33	90.75	90.05	90.33	90.05	90.05	90.19		95.37	91.59	94.95	94.11	100.00	100.00	92.01	95.37	95.79	94.95	95.79	91.59	94.95	94.53	94.53	93.69	94.11	94.53	94.11	93.69	95.79	94.53	93.69	92.01	93.69	92.01	92.85	93.27	94.53	93.27	92.01
CTLV-TL114	MH108984	90.47	90.75	91.03	91.31	90.75	91.17	90.75	90.75	90.89	96.63		93.69	94.95	97.47	95.37	95.37	94.11	100.00	98.73	98.31	99.57	92.85	94.95	96.21	96.21	94.95	95.37	95.79	95.79	94.95	97.47	96.21	95.37	94.11	94.95	94.11	94.11	94.53	96.63	94.53	94.11
CTLV-TL115	MH108985	92.01	96.07	96.35	94.95	96.07	97.89	96.07	96.07	92.57	89.91	90.75		94.11	92.43	91.59	91.59	97.47	93.69	94.53	92.85	93.69	93.27	94.95	96.21	96.21	94.53	95.79	95.79	95.79	94.53	93.27	95.79	95.79	99.15	95.37	94.95	94.53	94.95	97.05	94.95	95.37
CTLV-MTH	KC588948	97.89	91.45	91.73	92.99	91.73	91.73	91.73	91.73	92.71	90.33	91.17	91.59		94.11	94.95	94.95	94.95	94.95	95.79	94.53	95.37	92.43	98.73	97.05	97.05	96.21	96.21	96.63	96.63	96.21	94.53	96.63	96.21	94.53	95.37	94.11	96.21	95.37	97.05	94.11	94.53
CTLV-XHC	KC588947	89.77	90.05	90.33	90.33	90.05	90.47	90.05	90.05	89.91	95.65	98.17	90.05	90.47		94.11	94.11	92.85	97.47	97.05	97.05	97.89	91.59	94.11	95.37	95.37	94.11	94.53	94.95	94.95	94.11	95.79	95.37	94.53	92.85	93.69	92.85	93.27	93.69	95.37	93.27	92.85
CTLV-Pk	JX416228	90.19	90.05	90.33	90.75	90.05	90.33	90.05	90.05	90.19	100.00	96.63	89.91	90.33	95.65		100.00	92.01	95.37	95.79	94.95	95.79	91.59	94.95	94.53	94.53	93.69	94.11	94.53	94.11	93.69	95.79	94.53	93.69	92.01	93.69	92.01	92.85	93.27	94.53	93.27	92.01
CTLV-Ponkan8	KY706358	90.19	90.05	90.33	90.75	90.05	90.33	90.05	90.05	90.19	100.00	96.63	89.91	90.33	95.65	100.00		92.01	95.37	95.79	94.95	95.79	91.59	94.95	94.53	94.53	93.69	94.11	94.53	94.11	93.69	95.79	94.53	93.69	92.01	93.69	92.01	92.85	93.27	94.53	93.27	92.01
CTLV-ML	EU553489	91.59	99.15	99.43	96.35	100.00	96.21	100.00	100.00	91.59	90.05	90.75	96.07	91.73	90.05	90.05	90.05		94.11	94.53	93.27	94.11	93.69	94.95	96.21	96.21	94.53	95.79	95.79	95.79	94.53	93.69	95.79	96.21	98.31	94.53	94.95	94.53	94.95	97.05	94.95	95.37
CTLV-Kumquat1	AY646511	90.47	90.75	91.03	91.31	90.75	91.17	90.75	90.75	90.89	96.63	100.00	90.75	91.17	98.17	96.63	96.63	90.75		98.73	98.31	99.57	92.85	94.95	96.21	96.21	94.95	95.37	95.79	95.79	94.95	97.47	96.21	95.37	94.11	94.95	94.11	94.11	94.53	96.63	94.53	94.11
CTLV-LCd-NA-1	FJ355920	90.33	90.47	90.75	90.75	90.47	91.45	90.47	90.47	90.33	95.93	97.47	90.75	90.75	97.33	95.93	95.93	90.47	97.47		97.89	99.15	93.69	96.21	97.47	97.47	96.21	96.63	97.05	97.05	96.21	97.89	97.47	96.63	94.95	95.79	94.95	95.37	95.79	97.47	95.37	94.95
CTLV-Shatang Orange	JQ765412	90.33	90.33	90.61	90.89	90.33	90.75	90.33	90.33	90.47	96.21	98.45	90.33	91.03	98.31	96.21	96.21	90.33	98.45	97.61		98.73	92.01	94.53	95.79	95.79	94.53	94.95	95.37	95.37	94.53	97.05	95.79	94.95	93.27	94.95	93.69	93.69	94.11	95.79	93.69	93.27
CTLV-HJY	MH144341	90.47	91.17	91.45	91.45	91.17	91.59	91.17	91.17	90.75	96.63	99.01	91.17	91.17	98.59	96.63	96.63	91.17	99.01	98.17	98.87		92.85	95.37	96.63	96.63	95.37	95.79	96.21	96.21	95.37	97.89	96.63	95.79	94.11	94.95	94.11	94.53	94.95	96.63	94.53	94.11
CTLV-ASGV-1-HJY	MH144342	91.17	89.49	89.77	91.17	89.77	90.61	89.77	89.77	94.11	88.65	89.21	90.19	91.31	88.51	88.65	88.65	89.77	89.21	89.21	88.79	89.35		93.69	95.37	95.37	93.69	94.53	94.11	94.53	93.69	92.43	94.95	95.37	94.11	93.69	92.85	92.85	94.53	94.95	92.43	92.85
CTLV-ASGV-2-HJY	MH144343	98.17	90.75	91.03	92.71	91.03	91.45	91.03	91.03	91.59	89.49	90.19	91.59	96.91	89.49	89.49	89.49	91.03	90.19	90.05	90.05	90.19	90.33		97.47	97.47	96.63	97.47	97.05	97.05	96.63	94.53	97.05	96.63	95.37	96.21	94.53	97.05	96.63	97.47	95.37	94.95
CTLV-L	D16681	94.67	91.59	91.87	92.99	92.01	92.85	92.01	92.01	92.85	89.77	90.61	92.85	94.25	89.63	89.77	89.77	92.01	90.61	90.05	90.19	90.47	91.03	93.97		100.00	98.31	98.31	98.73	98.73	98.31	96.21	99.57	99.15	96.63	96.63	96.21	97.05	98.31	99.15	95.79	96.63
ASGV-Li-23	AB004063	94.39	91.87	92.15	93.27	92.29	93.13	92.29	92.29	92.85	89.77	90.61	93.13	94.25	89.91	89.77	89.77	92.29	90.61	90.33	90.47	90.75	91.31	93.97	99.71		98.31	98.31	98.73	98.73	98.31	96.21	99.57	99.15	96.63	96.63	96.21	97.05	98.31	99.15	95.79	96.63
ASGV-P-209	NC001749	92.29	90.05	90.33	92.15	90.33	90.33	90.33	90.33	92.29	89.35	89.49	90.19	91.87	88.79	89.35	89.35	90.33	89.49	89.21	89.35	89.49	90.19	92.15	92.15	92.43		97.47	97.89	97.05	100.00	94.95	98.31	97.47	94.95	95.37	94.53	96.21	96.63	97.47	94.53	94.95
ASGVp12	HE978837	92.29	91.03	91.03	92.29	90.61	91.03	90.61	90.61	91.31	89.35	89.49	91.03	91.59	89.07	89.35	89.35	90.61	89.49	89.77	89.63	89.91	88.93	92.01	92.71	92.71	90.33		99.57	97.89	97.47	95.37	97.89	97.47	96.21	96.21	95.37	96.63	98.31	98.31	95.79	95.79
ASGV-AC	KX988001	92.57	90.75	90.75	92.01	90.33	91.03	90.33	90.33	91.17	89.21	89.35	91.03	92.15	88.93	89.21	89.21	90.33	89.35	89.63	89.49	89.77	89.07	92.29	93.13	93.13	90.89	98.87		98.31	97.89	95.79	98.31	97.89	96.21	96.21	95.79	96.63	97.89	98.73	95.37	96.21
ASGV-HH	JN701424	92.15	91.03	91.31	92.43	90.89	91.73	90.89	90.89	91.59	90.05	90.47	91.31	92.01	90.05	90.05	90.05	90.89	90.47	90.47	90.33	90.89	90.05	91.73	93.55	93.55	91.87	91.59	92.43		97.05	95.79	98.31	97.89	96.21	96.21	95.79	96.63	97.05	98.73	95.37	96.21
ASGV-241KP	D14995	92.29	90.05	90.33	92.15	90.33	90.33	90.33	90.33	92.29	89.35	89.49	90.19	91.87	88.79	89.35	89.35	90.33	89.49	89.21	89.35	89.49	90.19	92.15	92.15	92.43	100.00	90.33	90.89	91.87		94.95	98.31	97.47	94.95	95.37	94.53	96.21	96.63	97.47	94.53	94.95
ASGV-Matsuco	LC084659	90.33	90.75	91.03	91.31	90.75	91.03	90.75	90.75	90.47	96.35	97.05	90.61	91.31	96.35	96.35	96.35	90.75	97.05	96.77	96.77	97.19	89.35	89.91	90.61	90.89	89.35	89.63	89.49	89.91	89.35		96.21	95.37	93.69	94.11	94.53	94.11	94.95	96.21	94.11	93.69
ASGV-FKSS2	LC143387	94.39	92.01	92.29	93.69	92.15	93.27	92.15	92.15	92.85	90.47	91.31	93.27	93.97	90.33	90.47	90.47	92.15	91.31	90.75	90.89	91.17	91.59	93.41	98.03	97.75	92.15	92.85	93.27	93.41	92.15	91.31		98.73	96.21	96.63	95.79	96.63	97.89	98.73	95.79	96.21
ASGV-N297	LC184610	93.69	91.87	92.15	93.27	92.01	93.13	92.01	92.01	92.71	89.91	90.47	93.13	93.55	89.77	89.91	89.91	92.01	90.47	90.19	90.33	90.61	92.01	93.27	97.61	97.89	91.73	92.01	92.71	92.71	91.73	90.75	97.89		96.63	95.79	95.37	97.05	97.47	98.31	94.95	95.79
ASGV-Kiyomi	LC184611	92.15	96.77	97.05	95.37	96.49	98.59	96.49	96.49	92.43	90.33	91.45	99.01	91.73	90.75	90.33	90.33	96.49	91.45	91.17	90.75	91.59	90.61	91.73	92.99	93.27	90.33	91.45	91.45	91.73	90.33	90.75	93.41	93.55		95.79	95.37	94.95	95.37	97.47	95.37	95.79
ASGV-Nagami	LC184612	94.39	90.05	90.33	92.15	90.33	91.59	90.33	90.33	92.57	89.35	90.47	91.45	94.67	90.05	89.35	89.35	90.33	90.47	91.03	90.61	90.75	91.87	93.41	93.13	93.13	91.45	90.61	91.31	91.59	91.45	89.21	92.85	92.99	91.87		94.11	95.37	95.37	97.05	94.53	94.53
ASGV-kfp	KR106996	91.31	90.75	91.03	90.89	91.17	91.17	91.17	91.17	90.61	88.65	89.77	91.87	91.45	89.49	88.65	88.65	91.17	89.77	89.77	90.05	90.19	89.49	90.19	91.17	91.45	90.05	89.49	90.05	90.33	90.05	89.91	91.45	91.03	91.73	91.31		94.95	94.53	97.05	94.53	97.05
ASGV-Ac	JX080201	92.57	90.89	91.17	92.15	91.03	91.87	91.03	91.03	91.03	89.63	90.75	91.45	92.85	90.33	89.63	89.63	91.03	90.75	90.47	90.89	91.17	89.35	92.01	93.13	93.41	90.33	92.01	92.15	91.73	90.33	90.33	93.83	93.41	91.87	91.87	92.01		95.79	97.05	94.11	95.37
ASGV-CHN	JQ308181	91.87	90.75	90.75	91.59	90.61	90.89	90.61	90.61	91.03	89.91	90.05	90.47	91.45	89.77	89.91	89.91	90.61	90.05	90.33	89.91	90.47	90.33	90.61	92.99	92.99	91.87	91.17	91.45	95.79	91.87	89.91	92.85	92.15	90.89	91.45	89.77	91.45		97.47	94.95	94.95
ASGV-YTG	KJ579253	93.13	92.01	92.29	93.69	91.87	92.85	91.87	91.87	93.55	91.03	91.31	92.43	93.27	90.89	91.03	91.03	91.87	91.31	92.15	91.17	91.73	91.59	92.99	93.69	93.69	92.85	92.99	93.27	96.07	92.85	91.45	93.83	93.13	92.57	92.99	91.31	92.71	94.95		96.63	97.47
ASGV-HT	KU947036	92.99	91.59	91.59	92.57	91.17	92.43	91.17	91.17	91.45	90.05	91.31	91.73	93.13	90.61	90.05	90.05	91.17	91.31	91.03	91.17	91.45	90.05	92.85	92.99	92.99	90.19	92.43	92.71	91.45	90.19	91.45	92.71	92.15	91.87	92.29	92.15	91.87	90.75	93.27		94.11
PBNLSV	AY596172	91.87	90.61	90.89	92.15	90.75	92.15	90.75	90.75	91.45	89.07	89.63	91.31	91.59	88.93	89.07	89.07	90.75	89.63	89.63	89.49	89.77	89.63	91.59	94.11	94.11	90.19	90.61	91.17	91.87	90.19	89.63	94.67	93.97	91.73	90.75	92.71	92.57	91.03	93.13	91.17	

CTLV: citrus tatter leaf virus; ASGV: apple stem grooving virus; PBNLSV: pear black necrotic leaf spot virus
