# Supplementary material for: Full genome characterization of 12 citrus tatter leaf virus isolates for the development of a detection assay
Source: PLoS One. 2019 Oct 17;14(10):e0223958. doi: 10.1371/journal.pone.0223958 (PMC6797102; doi:10.1371/journal.pone.0223958)
Supplement: S10 Table — (PDF) [file pone.0223958.s011.pdf]

S10 Table. Nucleotide (below diagonal) and amino acid (above diagonal) sequences identities (%) of movement protein (MP).

Isolate	Genbank	CTLV-IPPN122	CTLV-TL100	CTLV-TL101	CTLV-TL102	CTLV-TL103	CTLV-TL104	CTLV-TL110	CTLV-TL111	CTLV-TL112	CTLV-TL113	CTLV-TL114	CTLV-TL115	CTLV-MTH	CTLV-XHC	CTLV-Pk	CTLV-Ponkan8	CTLV-ML	CTLV-Kumquat1	CTLV-LCd-NA-1	CTLV-Shatang Orange	CTLV-HJY	CTLV-ASGV-1-HJY	CTLV-ASGV-2-HJY	CTLV-L	ASGV-Li-23	ASGV-P-209	ASGVp12	ASGV-AC	ASGV-HH	ASGV-241KP	ASGV-Matsuco	ASGV-FKSS2	ASGV-N297	ASGV-Kiyomi	ASGV-Nagami	ASGV-kfp	ASGV-Ac	ASGV-CHN	ASGV-YTG	ASGV-HT	PBNLSV
CTLV-IPPN122	MH108986		96.26	96.26	95.95	95.63	96.26	95.63	95.63	94.70	95.95	95.63	95.63	97.19	95.32	95.95	95.95	95.63	95.63	95.32	95.63	96.26	95.32	97.19	96.26	96.26	94.08	94.39	95.95	95.01	94.08	96.26	97.19	97.50	96.26	96.57	97.19	96.26	93.76	97.19	95.32	93.45
CTLV-TL100	MH108975	85.87		100.00	99.68	99.37	99.37	99.37	99.37	96.57	96.57	96.26	98.75	97.81	95.32	96.57	96.57	99.37	96.26	96.57	95.95	96.88	96.57	97.19	96.26	96.26	96.26	95.01	96.57	96.26	96.26	96.88	97.81	97.50	99.37	97.50	97.19	96.88	94.39	97.19	96.88	95.01
CTLV-TL101	MH108976	86.29	98.85		99.68	99.37	99.37	99.37	99.37	96.57	96.57	96.26	98.75	97.81	95.32	96.57	96.57	99.37	96.26	96.57	95.95	96.88	96.57	97.19	96.26	96.26	96.26	95.01	96.57	96.26	96.26	96.88	97.81	97.50	99.37	97.50	97.19	96.88	94.39	97.19	96.88	95.01
CTLV-TL102	MH108977	86.18	98.44	98.96		99.06	99.06	99.06	99.06	96.26	96.26	95.95	98.44	97.50	95.01	96.26	96.26	99.06	95.95	96.26	95.63	96.57	96.26	96.88	95.95	95.95	95.95	94.70	96.26	95.95	95.95	96.57	97.50	97.19	99.06	97.19	96.88	96.57	94.08	96.88	96.57	94.70
CTLV-TL103	MH108978	86.08	98.54	98.85	98.44		98.75	100.00	100.00	95.95	95.95	95.63	98.75	97.19	94.70	95.95	95.95	100.00	95.63	95.95	95.32	96.26	95.95	96.57	95.63	95.63	95.95	94.39	95.95	95.63	95.95	96.26	97.19	96.88	98.75	96.88	97.19	96.26	93.76	96.57	96.26	94.39
CTLV-TL104	MH108979	87.33	94.28	94.60	94.60	94.91		98.75	98.75	96.57	96.57	96.26	98.75	97.19	95.32	96.57	96.57	98.75	96.26	96.57	95.95	96.88	96.57	97.19	96.57	96.57	96.57	94.70	96.26	96.26	96.57	96.26	97.50	97.81	99.37	97.19	97.19	97.50	94.39	97.81	96.57	95.01
CTLV-TL110	MH108980	86.08	98.54	98.85	98.44	100.00	94.91		100.00	95.95	95.95	95.63	98.75	97.19	94.70	95.95	95.95	100.00	95.63	95.95	95.32	96.26	95.95	96.57	95.63	95.63	95.95	94.39	95.95	95.63	95.95	96.26	97.19	96.88	98.75	96.88	97.19	96.26	93.76	96.57	96.26	94.39
CTLV-TL111	MH108981	86.08	98.54	98.85	98.44	100.00	94.91	100.00		95.95	95.95	95.63	98.75	97.19	94.70	95.95	95.95	100.00	95.63	95.95	95.32	96.26	95.95	96.57	95.63	95.63	95.95	94.39	95.95	95.63	95.95	96.26	97.19	96.88	98.75	96.88	97.19	96.26	93.76	96.57	96.26	94.39
CTLV-TL112	MH108982	85.56	88.47	88.05	87.64	88.16	86.91	88.16	88.16		97.50	97.50	95.32	95.95	96.88	97.50	97.50	95.95	97.50	99.37	97.50	97.81	96.26	95.01	95.01	95.01	95.01	94.39	96.26	95.63	95.01	97.19	96.57	96.26	95.95	96.26	95.01	95.63	93.14	96.88	93.45	92.21
CTLV-TL113	MH108983	85.56	87.12	86.70	86.50	87.01	86.08	87.01	87.01	94.91		99.06	95.32	97.81	98.13	100.00	100.00	95.95	99.06	98.13	98.44	99.68	96.26	96.88	96.88	96.88	95.63	95.01	96.88	96.88	95.63	99.06	98.44	98.13	95.95	96.88	96.26	96.88	94.39	97.50	94.70	93.45
CTLV-TL114	MH108984	85.77	86.70	86.50	86.70	86.60	86.18	86.60	86.60	94.91	95.01		95.01	97.50	97.81	99.06	99.06	95.63	100.00	97.81	98.13	99.37	96.26	96.57	96.57	96.57	95.32	94.70	96.57	96.57	95.32	98.75	98.13	97.81	95.63	96.57	95.95	96.57	94.08	97.19	94.39	93.14
CTLV-TL115	MH108985	86.39	93.66	93.76	93.97	94.49	96.88	94.49	94.49	86.18	85.56	86.29		96.57	94.08	95.32	95.32	98.75	95.01	95.32	94.70	95.63	95.95	96.57	95.63	95.63	95.32	94.39	95.32	95.01	95.32	95.63	96.57	96.88	99.37	96.57	97.19	96.26	93.76	96.57	95.95	94.08
CTLV-MTH	KC588948	87.33	85.66	85.35	85.15	85.46	86.29	85.46	85.46	84.73	85.35	85.87	86.29		96.57	97.81	97.81	97.19	97.50	96.57	96.88	98.13	96.26	98.13	97.19	97.19	95.32	95.32	96.88	95.95	95.32	98.13	98.75	98.44	97.19	97.81	97.50	97.81	94.08	97.50	95.95	94.08
CTLV-XHC	KC588947	84.63	86.29	85.87	85.87	86.18	85.35	86.18	86.18	94.08	93.87	97.40	85.66	85.04		98.13	98.13	94.70	97.81	97.50	97.81	98.44	95.01	95.63	95.63	95.63	94.39	93.76	95.63	95.63	94.39	98.44	97.19	96.88	94.70	95.63	95.63	95.63	93.45	96.26	93.45	92.83
CTLV-Pk	JX416228	85.56	87.12	86.70	86.50	87.01	86.08	87.01	87.01	94.91	100.00	95.01	85.56	85.35	93.87		100.00	95.95	99.06	98.13	98.44	99.68	96.26	96.88	96.88	96.88	95.63	95.01	96.88	96.88	95.63	99.06	98.44	98.13	95.95	96.88	96.26	96.88	94.39	97.50	94.70	93.45
CTLV-Ponkan8	KY706358	85.56	87.12	86.70	86.50	87.01	86.08	87.01	87.01	94.91	100.00	95.01	85.56	85.35	93.87	100.00		95.95	99.06	98.13	98.44	99.68	96.26	96.88	96.88	96.88	95.63	95.01	96.88	96.88	95.63	99.06	98.44	98.13	95.95	96.88	96.26	96.88	94.39	97.50	94.70	93.45
CTLV-ML	EU553489	86.08	98.54	98.85	98.44	100.00	94.91	100.00	100.00	88.16	87.01	86.60	94.49	85.46	86.18	87.01	87.01		95.63	95.95	95.32	96.26	95.95	96.57	95.63	95.63	95.95	94.39	95.95	95.63	95.95	96.26	97.19	96.88	98.75	96.88	97.19	96.26	93.76	96.57	96.26	94.39
CTLV-Kumquat1	AY646511	85.77	86.70	86.50	86.70	86.60	86.18	86.60	86.60	94.91	95.01	100.00	86.29	85.87	97.40	95.01	95.01	86.60		97.81	98.13	99.37	96.26	96.57	96.57	96.57	95.32	94.70	96.57	96.57	95.32	98.75	98.13	97.81	95.63	96.57	95.95	96.57	94.08	97.19	94.39	93.14
CTLV-LCd-NA-1	FJ355920	85.87	88.47	88.05	87.64	88.16	86.91	88.16	88.16	99.58	95.32	95.11	86.18	85.04	94.49	95.32	95.32	88.16	95.11		97.81	98.44	96.26	95.63	95.63	95.63	95.63	95.01	96.88	96.26	95.63	97.81	97.19	96.88	95.95	96.88	95.63	96.26	93.76	97.50	94.08	92.21
CTLV-Shatang Orange	JQ765412	85.35	86.50	86.29	86.29	86.60	85.98	86.60	86.60	95.01	94.70	98.44	86.08	85.46	97.71	94.70	94.70	86.60	98.44	95.43		98.75	95.32	96.57	95.95	95.95	94.70	94.08	95.95	95.95	94.70	98.13	97.50	97.19	95.32	96.57	95.32	95.95	93.45	96.57	93.76	92.83
CTLV-HJY	MH144341	85.46	86.91	86.50	86.50	86.81	86.39	86.81	86.81	94.80	94.60	97.61	86.50	85.77	97.09	94.60	94.60	86.81	97.61	95.22	97.71		96.57	97.19	97.19	97.19	95.95	95.32	97.19	97.19	95.95	99.37	98.75	98.44	96.26	97.19	96.57	97.19	94.70	97.81	95.01	93.76
CTLV-ASGV-1-HJY	MH144342	84.42	86.18	86.08	86.08	85.98	87.33	85.98	85.98	84.63	84.83	85.46	86.81	84.31	85.25	84.83	84.83	85.98	85.46	84.63	84.83	84.83		96.26	95.95	95.95	95.32	94.70	96.57	96.26	95.32	96.57	96.88	97.19	96.57	96.57	96.26	95.95	94.70	96.88	95.01	92.83
CTLV-ASGV-2-HJY	MH144343	89.92	84.94	84.63	84.83	84.73	86.18	84.73	84.73	84.21	84.83	84.94	85.25	90.34	83.90	84.83	84.83	84.73	84.94	84.52	84.11	84.83	84.11		97.19	97.19	95.63	94.70	96.26	95.63	95.63	97.19	98.13	98.44	97.19	98.13	97.50	97.19	93.45	97.50	95.63	93.76
CTLV-L	D16681	84.63	84.83	84.73	84.52	84.83	84.11	84.83	84.83	83.07	85.04	84.11	84.21	84.63	83.28	85.04	85.04	84.83	84.11	83.28	83.59	83.90	83.80	85.66		98.75	94.70	94.39	96.26	95.01	94.70	96.88	98.13	98.44	96.26	96.88	96.57	96.57	93.14	96.88	95.01	93.45
ASGV-Li-23	AB004063	84.94	85.25	85.15	84.94	85.04	84.31	85.04	85.04	83.28	85.25	84.31	84.21	84.94	83.48	85.25	85.25	85.04	84.31	83.48	83.80	84.11	84.11	85.98	98.75		94.70	94.39	96.26	95.01	94.70	96.88	98.13	98.44	96.26	96.88	96.57	96.57	93.14	96.88	95.01	93.45
ASGV-P-209	NC001749	87.43	85.25	84.52	84.73	84.83	85.87	84.83	84.83	85.66	85.35	86.08	85.46	91.58	85.35	85.35	85.35	84.83	86.08	85.98	85.56	86.08	84.00	90.23	84.73	85.15		94.08	96.26	96.26	100.00	95.32	95.95	95.95	95.95	95.95	95.01	95.32	94.08	96.26	95.01	92.83
ASGVp12	HE978837	84.11	85.66	85.56	85.46	85.66	85.04	85.66	85.66	86.29	86.18	87.01	85.77	84.21	86.81	86.18	86.18	85.66	87.01	86.60	86.70	86.81	84.11	83.28	83.80	83.59	84.83		97.81	95.01	94.08	95.01	95.63	95.32	95.01	95.01	95.32	94.70	93.76	96.26	94.08	93.14
ASGV-AC	KX988001	84.83	86.29	85.98	85.87	86.08	85.87	86.08	86.08	86.81	86.60	87.12	85.98	85.04	86.91	86.60	86.60	86.08	87.12	87.12	86.60	86.70	85.25	83.90	84.11	84.11	85.66	97.50		97.19	96.26	96.88	97.50	97.19	95.95	97.19	96.88	96.26	95.32	97.81	95.63	94.39
ASGV-HH	JN701424	84.52	84.42	84.00	84.21	83.80	84.94	83.80	83.80	83.90	85.35	84.52	84.63	84.83	83.17	85.35	85.35	83.80	84.52	84.21	84.11	84.42	83.80	85.35	87.64	87.43	86.50	83.80	84.11		96.26	96.57	96.57	96.26	95.63	95.63	95.95	95.63	96.88	96.57	95.63	93.45
ASGV-241KP	D14995	87.43	85.25	84.52	84.73	84.83	85.87	84.83	84.83	85.66	85.35	86.08	85.46	91.58	85.35	85.35	85.35	84.83	86.08	85.98	85.56	86.08	84.00	90.23	84.73	85.15	100.00	84.83	85.66	86.50		95.32	95.95	95.95	95.95	95.95	95.01	95.32	94.08	96.26	95.01	92.83
ASGV-Matsuco	LC084659	84.94	87.33	86.91	86.70	87.43	86.29	87.43	87.43	95.43	96.57	94.91	86.39	85.46	94.80	96.57	96.57	87.43	94.91	95.84	95.32	95.11	84.94	84.52	83.80	84.00	85.66	87.01	87.33	84.42	85.66		98.44	98.13	96.26	97.19	96.57	96.57	94.39	97.19	95.01	93.45
ASGV-FKSS2	LC143387	85.87	86.29	85.98	85.98	86.08	86.08	86.08	86.08	84.11	85.25	85.35	85.66	86.60	84.31	85.25	85.25	86.08	85.35	84.31	84.63	85.15	84.31	86.91	85.84	86.26	86.91	84.73	85.04	88.57	86.91	84.42		99.68	97.19	98.13	97.50	97.50	94.70	97.81	95.95	94.39
ASGV-N297	LC184610	85.46	86.08	85.77	85.35	85.66	85.66	85.66	85.66	84.11	85.04	85.25	85.25	86.08	84.42	85.04	85.04	85.66	85.25	84.31	84.73	85.04	85.15	86.70	94.80	95.43	86.70	85.25	85.77	88.99	86.70	84.83	96.05		97.50	98.13	97.81	97.81	94.39	98.13	95.95	94.39
ASGV-Kiyomi	LC184611	86.91	94.18	94.49	94.49	95.01	97.61	95.01	95.01	86.81	85.56	86.29	98.65	86.39	85.66	85.56	85.56	95.01	86.29	86.81	86.08	86.50	87.22	85.77	84.52	84.52	85.56	85.87	86.70	84.73	85.56	86.39	85.98	85.35		97.19	97.19	96.88	94.39	97.19	96.57	94.70
ASGV-Nagami	LC184612	87.12	85.98	85.66	85.46	86.18	86.60	86.18	86.18	85.87	85.25	85.35	86.39	92.52	84.31	85.25	85.25	86.18	85.35	85.98	85.15	85.25	84.73	91.38	84.52	84.63	92.93	83.59	84.63	85.87	92.93	85.25	86.18	86.29	86.70		96.88	96.57	93.76	98.13	95.32	93.76
ASGV-kfp	KR106996	84.52	84.42	84.52	84.42	84.63	85.46	84.63	84.63	83.17	84.21	83.90	85.66	84.42	83.17	84.21	84.21	84.63	83.90	83.38	83.48	84.00	84.83	84.00	83.48	83.69	83.07	85.66	86.60	83.90	83.07	84.83	83.69	84.63	85.35	83.69		97.19	94.08	97.50	96.26	94.39
ASGV-Ac	JX080201	87.01	86.18	85.98	85.66	86.29	86.39	86.29	86.29	85.25	85.15	85.87	85.25	86.91	85.15	85.15	85.15	86.29	85.87	85.56	85.87	86.08	85.77	85.66	84.42	84.63	85.77	85.15	85.77	83.28	85.77	85.25	85.25	85.87	85.46	86.81	84.00		93.76	97.19	95.32	93.14
ASGV-CHN	JQ308181	84.21	83.69	83.48	83.48	83.07	84.52	83.07	83.07	82.24	84.11	83.07	84.31	84.31	81.82	84.11	84.11	83.07	83.07	82.55	82.65	83.17	82.86	84.83	86.08	85.77	86.18	83.07	83.38	94.49	86.18	83.07	87.53	87.64	84.42	85.15	82.65	84.11		94.70	93.76	92.21
ASGV-YTG	KJ579253	86.18	86.60	86.50	86.39	86.18	87.01	86.18	86.18	86.18	86.60	86.70	86.08	85.56	86.08	86.60	86.60	86.18	86.70	86.39	86.18	85.87	85.56	86.08	85.56	85.98	85.87	86.29	86.81	85.98	85.87	86.08	86.70	86.70	86.60	85.87	85.77	86.91	84.11		95.63	94.39
ASGV-HT	KU947036	85.66	84.73	84.94	84.52	84.94	84.94	84.94	84.94	84.00	83.80	83.17	84.73	83.17	82.03	83.80	83.80	84.94	83.17	84.21	82.76	83.07	82.86	83.80	83.17	82.86	83.59	83.90	85.15	84.00	83.59	83.80	83.28	83.48	84.73	83.69	83.48	82.65	83.17	83.69		93.76
PBNLSV	AY596172	84.83	84.73	84.94	84.83	84.73	85.04	84.73	84.73	84.31	84.42	85.35	85.25	84.00	84.63	84.42	84.42	84.73	85.35	84.31	84.83	84.94	84.00	84.73	83.07	83.28	84.73	87.43	87.64	82.86	84.73	83.90	83.28	84.42	85.35	84.11	85.56	84.63	82.34	86.39	83.17	

CTLV: citrus tatter leaf virus; ASGV: apple stem grooving virus; PBNLSV: pear black necrotic leaf spot virus
