# Supplementary material for: Full genome characterization of 12 citrus tatter leaf virus isolates for the development of a detection assay
Source: PLoS One. 2019 Oct 17;14(10):e0223958. doi: 10.1371/journal.pone.0223958 (PMC6797102; doi:10.1371/journal.pone.0223958)
Supplement: S1 Table — (PDF) [file pone.0223958.s002.pdf]

Isolate	GenBank Accession No.	Partial sequence of citrus tatter leaf virus
ASGV-P-209* (apple)	NC001749	GGTTTTCGAGGCAGGTTCGGAAAGTAACCTGGAACTGGAGGGTTAGGAGTCGTGTGAAATTCCGCAAACTTGGTCGCGGTCTTGCAGGTTGAC
CTLV-IPPN122	MH108986	.....................G....................C..........................T........................
CTLV-TL100	MH108975	.....................G...A........................
CTLV-TL101	MH108976	.....................G........................A......................A........................
CTLV-TL102	MH108977	.....................G...A........................
CTLV-TL103	MH108978	.....................G...G........................
CTLV-TL104	MH108979	.....................G...G.A........................
CTLV-TL110	MH108980	.....................G...G........................
CTLV-TL111	MH108981	.....................G...G........................
CTLV-TL112	MH108982	.....................G...............................A.A..........G..........................
CTLV-TL113	MH108983	............G........G......T........................T.A.....................................
CTLV-TL114	MH108984	.....................G....G.T........................T.A............T.............A..........
CTLV-TL115	MH108985	.....................G...G.A........................
CTLV-MTH	KC588948	.....................G....................C..........................T........................
CTLV-XHC	KC588947	.....................G....G.T........................T.A............T........................
CTLV-Pk	JX416228	............G........G......T........................T.A.....................................
CTLV-Ponkan8	KY706358	............G........G......T........................T.A.....................................
CTLV-ML	EU553489	.....................G...G........................
CTLV-Kumquat1	AY646511	.....................G....G.T........................T.A............T.............A..........
CTLV-LCd-NA-1	FJ355920	......T..............G......T........................T.A.....................................
CTLV-Shatang Orange	JQ765412	.....................G....G.T........................T.A............T........................
CTLV-HJY	MH144341	.....................G....G.T........................T.A............T........................
CTLV-ASGV-1-HJY	MH144342	......T..............G...G.T........................
CTLV-ASGV-2-HJY	MH144343	.....................G....................C............A..........G.T........................
ASGV-Matsuco	LC084659	......T..............G......T........................T.A.....................................
ASGV-FKSS2	LC143387	.....................G...
ASGV-N297	LC184610	......T.....G........G...
ASGV-Kiyomi	LC184611	.....................G...G.A........................
ASGV-Nagami	LC184612	............G........G.....T...T........................
CTLV 6315 F1		CGAGGCAGGTTCGGAGAGTA
CTLV 6314 F2		GAGGCAGGTTCGGAGAGTA
CTLV 6314 F3		TGAGGCAGGTTCGGAGAGTAA
CTLV 6337 P FAM		TGGAACTGGAGGGTTAG
CTLV R		GGTCGCGGTCTTGCAGG

S1 Fig. Citrus tatter leaf virus detection assay targeting region. Multiple nucleotide sequences alignment of citrus tatter leaf virus and apple stem grooving virus isolated from citrus and citrus relatives host. Citrus tatter leaf virus detection assay targeting region (highlighted in dark grey) and primers-probe set are also shown. Apple stem grooving virus isolate P-209 is used here to represent the species.
